# CYP3A triggers BDE47-induced ferritinophagy and ferroptosis in spermatogenic cells through ROS-mediated m^6^A regulation of ATG12

**DOI:** 10.7555/JBR.40.20260159

**Published:** 2026-07-28

**Authors:** Shuyu Xu, Wenxia Fan, Jiangxue Qian, Yuewen He, Chunjin Li, Chao Chen, Li Wang, Yongquan Yu, Chao Wang, Shoulin Wang

**Affiliations:** 1 State Key Lab of Reproductive Medicine and Offspring Health, Institute of Toxicology, Nanjing Medical University, Nanjing, Jiangsu 211116, China; 2 Key Lab of Modern Toxicology of Ministry of Education, Center for Global Health, School of Public Health, Nanjing Medical University, Nanjing, Jiangsu 211116, China

**Keywords:** cytochrome P450 3A, 2,2',4,4'-tetrabromodiphenyl ether, reactive oxygen species, ferritinophagy, N6-methyladenosine, male reproductive damage

## Abstract

Cytochrome P450 CYP3A (CYP3A) is among the most abundant hepatic cytochrome P450 subfamilies and also mediates the metabolism and toxicity of xenobiotics. Previous studies have reported that CYP3A is also expressed in the testis; however, its role and molecular mechanism in mediating male reproductive damage remain unclear. In this study, through *in vitro* and *in vivo* experiments, we demonstrated the role of CYP3A in 2,2',4,4'-tetrabromodiphenyl ether (BDE47)-induced reproductive toxicity. The results showed that BDE47 induced CYP3A expression in mouse testes, leading to oxidative stress and ferroptosis through excessive reactive oxygen species (ROS) and ferrous iron (Fe^2+^) overload, which was demonstrated by CYP3A overexpression or knockdown experiments in GC-2 cells. Mechanistically, in addition to direct ROS generation during metabolic processing, ferritinophagy contributed to intracellular Fe^2+^ accumulation. Specifically, BDE47-induced ROS was associated with reduced N6-methyladenosine (m^6^A) modification of *Atg12* mRNA and increased autophagy-related (ATG12) expression, thereby promoting ferritin heavy chain 1 (FTH1) degradation and subsequent Fe^2+^ overload. These results were further validated by experiments using hydrogen peroxide or antioxidants in GC-2 cells, as well as by *Atg12* haploinsufficiency in mice. Our findings demonstrate that CYP3A plays a critical role in male reproductive toxicity induced by BDE47.

## Introduction

Epidemiological data indicate that 8%–12% of couples worldwide experience infertility, with male factors accounting for 30%–50% of cases^[1]^. Although congenital anomalies, endocrine disorders, and aging contribute to this condition, mounting evidence highlights environmental toxicants as a major modifiable risk factor^[2]^. Polybrominated diphenyl ethers (PBDEs), as typical environmental endocrine-disrupting chemicals (EDCs), have been restricted and phased out for several years, yet large amounts of PBDE-containing commercial products remain in use and are discarded annually. To date, significant concentrations of PBDEs are still detectable in the environment and in various human biological samples, including blood and breast milk^[3–4]^. Among these, 2,2',4,4'-tetrabromodiphenyl ether (BDE47), a predominant PBDE congener that is readily enriched in organisms, can be detected in various biological samples. Studies have shown that BDE47 reaches an average level of 43 ng/g dry weight in human hair, 38 ng/g dry weight in nails, and 20 ng/g lipid weight in serum^[5]^. Although BDE47 has been banned, it continues to accumulate in the environment and the human body, where it exerts toxic effects. Studies have demonstrated its detrimental effects on male reproduction, including disrupted spermatogenesis, reduced sperm motility, and testicular atrophy^[6]^. BDE47 exerts toxic effects on spermatogenic cells, particularly on spermatocytes—a type of germ cell in the testis^[7]^. Previous studies have shown that BDE47 induces spermatocyte apoptosis through mechanisms involving cytochrome P450 3A (CYP3A)-mediated metabolic activation and oxidative stress; however, the underlying molecular mechanism remains unclear^[8–9]^.

The cytochrome P450 (CYP450) superfamily represents an important group of phase Ⅰ metabolic enzymes that dominate xenobiotic oxidation and catalyze over 90% of drug biotransformation^[10]^. Among this superfamily, the CYP3A subfamily stands out as the most pharmacologically and toxicologically significant isoform due to its dual prominence: it constitutes 30%–40% of total hepatic CYP450 enzymes and exhibits remarkable substrate promiscuity toward a wide range of structurally diverse lipophilic compounds, including 30% of clinical drugs and various environmental toxicants^[11–12]^. This metabolic versatility arises from CYP3A's uniquely flexible active site architecture, which accommodates bulky substrates through conformational plasticity^[12]^. Consequently, CYP3A is a major enzyme involved in the metabolism of many xenobiotics. Previous studies have shown that CYP3A is not only highly expressed in the liver, but is also expressed in extrahepatic organs such as the testes; however, its extrahepatic expression has received less attention^[8]^. Extrahepatic CYP-mediated metabolic activation may amplify tissue-specific toxicity through dual mechanisms: (1) production of electrophilic metabolites, and (2) overgeneration of reactive oxygen species (ROS) that overwhelms cellular antioxidant defenses^[13–14]^. CYP3A's capacity to concurrently generate ROS and modulate iron redox cycling (*via* FeO^3+^ intermediates) positions it as a potential master regulator of phospholipid peroxidation, one of the biochemical hallmarks of ferroptosis^[15–17]^.

Ferroptosis is a distinct form of regulated cell death driven by iron-dependent phospholipid peroxidation, and it is intricately regulated by interconnected metabolic pathways that include redox homeostasis, iron metabolism, mitochondrial bioenergetics, and nutrient processing^[18]^. Previous studies have shown that ROS generated by NADPH-cytochrome P450 reductase (POR) and NADH-cytochrome b5 reductase (CYB5R1) contribute to phospholipid peroxidation during ferroptosis^[19]^. The interaction between reductases and CYPs, especially CYP3A, is inseparable. However, while current studies have described the role of oxidoreductases in ferroptosis, the involvement of CYPs, particularly CYP3A, remains less understood. Therefore, we propose that byproducts generated by CYP3A-mediated metabolism act as an upstream trigger for ferroptosis, synergizing iron dyshomeostasis with lipid peroxide accumulation in germ cells.

Ferroptosis is known to be regulated through multiple layers of control, encompassing epigenetic, transcriptional, post-transcriptional, and post-translational mechanisms such as N6-methyladenosine (m^6^A) modification^[20]^. A key autophagic process implicated in ferroptosis is ferritinophagy—a selective autophagic process that degrades iron-storage ferritin complexes through nuclear receptor coactivator 4 (NCOA4)-mediated recognition of ferritin heavy chain 1 (FTH1)^[21]^. This process leads to an increase in intracellular labile iron, thereby promoting the accumulation of lipid peroxides, a central event in the execution of ferroptosis^[22]^. As an important epigenetic modification, m^6^A modification has potential roles in regulating ferritinophagy^[23]^. ROS, m^6^A modification, and ferritinophagy are closely interlinked in the toxic effects of exogenous chemicals.

Based on the above background, this study aims to investigate the role of CYP3A-mediated oxidative metabolism in ferroptosis and its contribution to xenobiotic-induced reproductive damage. Specifically, using both *in vitro* and *in vivo* models, we adopted a ferritinophagy-centered approach to examine how CYP3A-mediated oxidative metabolism disrupts iron and redox homeostasis, thereby promoting ferroptosis in germ cells. This study seeks to provide insights into the mechanistic link between CYP3A-mediated metabolism, oxidative stress, and ferroptosis, ultimately advancing the understanding of xenobiotic-induced male infertility.

## Materials and methods

### Reagents

BDE47 was purchased from Macklin Inc. (Cat. #O885862, Shanghai, China). Hydrogen peroxide (H_2_O_2_) was purchased from Sigma (Cat. #H1009, St. Louis, MO, USA). N-Acetylcysteine (NAC) was obtained from MedChemExpress (Cat. #HY-B0215, Shanghai, China). Deferiprone (DFP) was purchased from MedChemExpress (Cat. #HY-B0568). Antibody information is shown in ***Supplementary Table 1***.

### Experimental animals

All animal procedures were approved by the Institutional Animal Care and Use Committee of Nanjing Medical University and conducted in accordance with the NIH Guide for the Care and Use of Laboratory Animals (License Number: SYXK (Su) 2020-0006; Ethics Number: 2003001). Wild-type C57BL/6 male mice (22–25 g) were obtained from Nanjing Medical University. *Atg12*^+/−^ mice on a C57BL/6 background were generated by Bangyao Bio (Shanghai, China) using CRISPR-Cas9 technology. Cas9 mRNA and sgRNAs were microinjected into fertilized zygotes to induce targeted gene editing. The mice were housed in a specific pathogen-free facility under controlled conditions (temperature: 20–26 ℃; humidity: 30%–70%; 12-h light/dark cycle) with *ad libitum* access to sterile food and water.

According to existing literature, the average BDE47 content in human serum is approximately 20 ng/g lipid, ranging from 4.3 to 240 ng/g lipid^[5]^. Based on previously reported human exposure levels, published experimental studies, and our previous pharmacokinetic/toxicological investigations of BDE47^[8,24]^, a dose of 1.5 mg/kg was selected to model a relatively high BDE47 exposure level. Mice received oral gavage as follows: the BDE47 group received 1.5 mg/kg BDE47 (dissolved in DMSO and diluted with sterile saline) *via* daily oral gavage (6 days/week) for 8 weeks. The control group received equivalent volumes of sterile saline with an equal amount of DMSO. All experiments were performed with five mice per group, randomly assigned to either the control or experimental group.

### Cell culture and treatment

GC-2 spermatocyte cells (ATCC, Cat. #CRL-2196, Manassas, VA, USA) were maintained in Dulbecco's Modified Eagle Medium (DMEM; Cat. #BC-M-005, Biochannel, Nanjing, China) supplemented with 10% (v/v) heat-inactivated fetal bovine serum (FBS; Cat. #BC-SE-FBS01, Biochannel), 100 U/mL penicillin, and 100 μg/mL streptomycin (Cat. #C0222, Beyotime, Shanghai, China). Cells were cultured at 37 ℃ in a humidified 5% CO_2_ atmosphere and passaged at 80%–90% confluence. Based on cell viability results, GC-2 cells were treated with 0, 15, and 30 μmol/L BDE47 for 48 h. In the rescue experiments, GC-2 cells were treated with BDE47 (30 μmol/L) alone or combined with NAC (5 mmol/L) or DFP (1 μmol/L) for 48 h. In the hydrogen peroxide (H_2_O_2_) experiments, GC-2 cells were treated with 0, 25, and 50 μmol/L H_2_O_2_ for 48 h.

### Cell transfection

Small interfering RNA (siRNA) targeting *Alkbh5* (si*Alkbh5*) and *Cyp3a11* (si*Cyp3a11*) and negative control (NC) siRNA (siNC) were designed and synthesized by Hanbio Biotechnology (Hanbio, Shanghai, China). The siRNA duplex sequences (sense/antisense) are as follows (5*'*–3*'*): siNC, UUCUCCGAACGUGUCACGUTT/ACGUGACACGUUCGGAGAATT; si*Cyp3a11*, GGUUCAGCAAGGAGAACAATT/UUGUUCUCCUUGCUGAACCTT; si*Alkbh5*, GGAAAUGGACAAAGAAGAATT/UUCUUCUUUGUCCAUUUCCTT. Briefly, GC-2 cells were seeded into 6-well plates and cultured to 60% confluence. Prior to transfection, cells were starved for 2 h in DMEM without FBS or antibiotics. The transfection mixture was prepared as follows: a) 0.5 mL DMEM; b) 0.5 mL DMEM containing 50 nmol/L of siRNA (per well); c) 0.5 mL DMEM containing 2.5 μL Lipofectamine 2000 Transfection Reagent (Cat. #TL201-01, Vazyme, Nanjing, China). Solutions b and c were each mixed gently and incubated at room temperature for 5 min, then combined and incubated for an additional 15 min. The combined mixture (b + c) was then mixed with solution A and added to the GC-2 cells. Transfection was carried out for 6 h, after which the medium was replaced with complete culture medium. The medium was replaced again with fresh complete medium 24 h post-transfection.

The primers used to amplify the *CYP3A4* coding sequence for plasmid construction were (5*'*–3*'*): CTAGACTAGTCACCATGGCTCTCATCCCAGACTTGGCCA/GATCTGATCAGTGGTACCGAGAGTAGGGTCTGAACCGGT. Subsequently, the recombinant plasmid pLVX *CYP3A4*-mCMV-ZsGreen containing the target gene *CYP3A4* was introduced into 293T cells to produce a high-titer lentivirus. The lentiviral supernatant was collected at 48 and 72 h post-transfection, pooled, filtered through a 0.45 μm filter, and concentrated by ultracentrifugation at 50000 *g* for 2 h at 4℃. The viral pellet was resuspended in phosphate-buffered saline (PBS), and the viral titer was determined by qPCR to be approximately 1 × 10^8^ TU/mL. For lentiviral transduction, GC-2 cells were seeded in 6-well plates at 60% confluence and incubated with the lentivirus at a multiplicity of infection of 10 in the presence of 8 μg/mL polybrene. After 24 h of incubation, the medium was replaced with fresh complete medium. Stable *CYP3A4*-overexpressing cells (*CYP3A4*-OE) were obtained after 72 h of transduction, followed by selection with 2 μg/mL puromycin for 7 days.

### Cell viability assay

Cell viability was assessed using a cell counting kit-8 (CCK-8; Cat. #C0038, Beyotime). Briefly, 2 × 10^3^ cells/well were seeded into 96-well plates and allowed to adhere for 24 h. Cells were treated with BDE47 alone (0, 2.5, 5, 10, 20, 40, 80, 160, and 320 μmol/L) or in combination with 1 μmol/L deferiprone (DFP) for 48 h. After treatment, cells were washed twice with PBS (1×, pH 7.4), and 100 μL of CCK-8 reagent (10% in serum-free DMEM) was added to each well. Following a 2-h incubation at 37 ℃, absorbance was measured at 450 nm using an EnSpire Multimode Plate Reader (PerkinElmer, Waltham, MA, USA). The results are presented as mean ± standard deviation (SD) of six samples.

### Computer-assisted sperm analysis (CASA)

For sperm collection and analysis, 400 μL of PBS was placed in a 1.5 mL Eppendorf tube, labeled, and pre-warmed in a 37 ℃ water bath. After euthanasia, the lower abdominal wall of exposed mice was incised, and the entire right epididymis was removed. The cauda epididymidis was then dissected, and a small incision of approximately 1 mm was made using ophthalmic scissors. The tissue was placed into a pre-warmed EP tube containing PBS and incubated at 37 ℃ for 5 min to allow free dispersion of spermatozoa. Following gentle pipetting to mix the suspension, 10 μL of the sample was loaded onto a round glass slide for a CASA system. A coverslip was applied, and fifteen different fields per sample were selected to record parameters including sperm count and motility.

### Histological analysis

Testicular tissues were fixed in Davidson's fixative for 24 h at 4 ℃. All samples underwent dehydration in graded ethanol, clearing in xylene, and paraffin embedding. Sections (5 μm thickness) were cut using a rotary microtome (Leica RM2235, Wetzlar, Germany) and stained with hematoxylin (5 min) and eosin (2 min) for histological evaluation. Slides were imaged under a light microscope (Nikon Eclipse E100, Nikon, Tokyo, Japan).

### Ultrastructure observation

Cells were cultured for 48 h before the experiment, and samples were fixed with 2.5% glutaraldehyde, rinsed with 0.1 mol/L phosphate buffer, and subsequently fixed with 1% osmium tetroxide. After another rinse, samples were dehydrated in acetone and embedded in an embedding agent. Ultrathin sections were stained with lead citrate. Ultrastructural micrographs were acquired using a JEM-1400Flash transmission electron microscope (JEOL, Tokyo, Japan).

### RNA extraction and reverse transcription-quantitative PCR (RT-qPCR)

Total RNA was isolated using RNA Isolater Total RNA Extraction Reagent (Cat. #R401-01, Vazyme) according to the manufacturer's protocol. The concentration and quality of RNA were assessed using a NanoDrop (ThermoFisher, Shanghai, China), with an optical density 260/280 ratio falling within the range of 1.8–2.0 deemed acceptable. Complementary DNA (cDNA) was synthesized using HiScript Ⅱ Q RT Supermix for qPCR (Cat. #R222-01, Vazyme). To analyze the relative mRNA expression levels, RT-qPCR was performed using ChamQ Universal SYBR qPCR Master Mix (Cat. #Q711-02, Vazyme) on a LightCycler 96 (Roche, Beijing, China). The reaction program was as follows: pre-denaturation at 95 ℃ for 30 s; 40 cycles of denaturation at 95 ℃ for 10 s and annealing at 60 ℃ for 30 s; 95 ℃ for 15 s; 60 ℃ for 60 s; 95 ℃ for 15 s, followed by cooling at 37 ℃ for 30 s. *Gapdh* was used as the internal control, and relative mRNA expression levels were calculated using the 2^–ΔΔCt^ method. The primer sequences used in this article were synthesized by Generay (Shanghai, China) (5′–3′): *Alkbh5*-F, ACGTTGACCCCATCCACATC; *Alkbh5*-R, AATGTCCTGAGGCCGTATGC; *Atg12*-F, TCCTTAAACTGGTGGCCTCG; *Atg12*-R, CTCTTCCCACAGCACCGAAA; *Gapdh*-F, AACTTTGGCATTGTGGAAGGG; *Gapdh*-R, GACACATTGGGGGTAGGAACA.

### Western blotting analysis

Tissues and cells were lysed in RIPA buffer (Cat. #P0013, Beyotime) containing protease and phosphatase inhibitors (Cat. #04693132001, Roche). Protein concentrations were quantified using the BCA assay (Cat. #E112, Vazyme) with a standard curve. Equal amounts of protein (30 μg per lane) were separated on 10% SDS-PAGE gels and transferred to nitrocellulose membranes (0.45 μm; Cat. #10600002, GE Healthcare, Shanghai, China). Membranes were blocked with 5% non-fat milk in TBST (Tris-buffered saline with 0.1% Tween-20) for 1 h at room temperature, then incubated overnight at 4 ℃ with primary antibodies. After three TBST washes, membranes were probed with HRP-conjugated secondary antibodies (1∶2000; AB0101, Abways, Shanghai, China) for 1 h at room temperature. Bands were visualized using an ECL kit (Cat. #E411-05, Vazyme) on a Tanon 5200 Chemiluminescent Imaging System. All bands were quantified using ImageJ software (NIH, USA). Antibody information is shown in ***Supplementary Table 1***.

### Dot blotting analysis

Total RNA was extracted using RNA Isolater reagent. RNA concentrations were adjusted to 500, 250, and 125 ng/μL with RNase-free water. Samples were denatured at 95 ℃ for 3 min, snap-cooled on ice, and spotted onto nitrocellulose membranes (Cat. #RPN303B, GE Healthcare). Membranes were UV cross-linked (120 mJ/cm^2^; CX-2000, UVP), stained with 0.05% methylene blue (5 min), and destained in RNase-free water. After blocking with 5% non-fat milk, membranes were incubated with an anti-m^6^A antibody overnight at 4 ℃, followed by an HRP-conjugated secondary antibody. Signal detection and normalization were performed according to the Western blotting protocol.

### Immunofluorescence (IF) analysis

Tissue sample processing: Testicular tissues were harvested from mice and immediately fixed in 4% paraformaldehyde (PFA) for 24 h at 4 ℃. All samples underwent dehydration in graded ethanol, clearing in xylene, and paraffin embedding. Sections (5 μm thickness) were cut using a rotary microtome (Leica RM2235). For antigen retrieval, sections were heated in sodium citrate buffer (10 mmol/L, pH 6.0) at 95 ℃ for 15 min, then cooled to room temperature.

Samples (tissue sections or cultured cells) were permeabilized with 0.2% Triton X-100 (Cat. #P0096, Beyotime) in 1× PBS for 15 min at room temperature, then blocked with 3% bovine serum albumin in 1× PBS for 1 h at 37 ℃. For cultured cells, GC-2 cells treated with BDE47 or H_2_O_2_ were fixed with 4% PFA for 30 min at room temperature prior to permeabilization. Primary antibodies (specific to the target proteins) were diluted in blocking buffer (without Triton X-100) and applied to samples overnight at 4 ℃. After three washes with 1× PBS (5 min each), samples were incubated with fluorophore-labeled secondary antibodies for 1 h at 37 ℃. Following three additional washes with 1× PBS, nuclei were stained with DAPI (Cat. #C1002, Beyotime) for 15 min at room temperature. All samples were photographed using a fluorescence microscope (LSM700, Zeiss, Germany). Fluorescence intensity was quantified using ImageJ software. The average fluorescence intensity of three independent samples was analyzed.

### Immunofluorescence co-localization staining

Immunofluorescence co-localization staining was performed using a double-label three-color multiplex fluorescence staining kit (AiFang Biological, Changsha, China). All procedures were carried out according to the manufacturer's instructions. Images were acquired using a fluorescence microscope (LSM700). Fluorescence intensity was quantified using ImageJ software. The average fluorescence intensity of three independent samples was analyzed.

### Detection of ROS, Fe^2^^+^, and lipid peroxide (LPO) levels in cells

The levels of ROS, Fe^2+^, and LPO in living cells were detected using fluorescent probes according to the manufacturer's protocols: dihydroethidium (DHE; Cat. #S0063, Beyotime) for ROS detection, FerroOrange (Cat. #F374, DOJINDO, Shanghai, China) for Fe^2+^ detection, and Liperfluo (Cat. #L248, DOJINDO) for LPO assessment. Cells were incubated with the respective probes for the recommended time and then analyzed using a fluorescence microscope (LSM700). Fluorescence intensity was measured using ImageJ software. For each of three independent samples, three random fields of view were analyzed.

### Assessment of glutathione (GSH), superoxide dismutase (SOD) activities, and malondialdehyde (MDA) levels

The levels of GSH, SOD activity, and MDA in cells and mouse tissues were assessed following the manufacturer's protocols using the following kits: GSH and GSSG Assay Kit (Cat. #S0053, Beyotime), Total Superoxide Dismutase Activity Assay Kit (Cat. #S0109, Beyotime), and Malondialdehyde Assay Kit (Cat. #S0131S, Beyotime). Samples were prepared according to the kit instructions, and absorbance was measured using a microplate reader. Protein content was quantified using the BCA assay with a standard curve. Finally, levels were standardized based on the protein content of each sample.

### Tissue iron levels

Iron concentrations in mouse tissues were determined using the Iron Assay Kit-Colorimetric (Cat. #I291, DOJINDO) according to the manufacturer's protocol. Samples were homogenized, and the resulting supernatants were analyzed for iron content. Tissues were uniformly weighed before sample preparation, and the results were standardized by tissue weight.

### m^6^A-specific methylated RNA immunoprecipitation-qPCR (MeRIP-qPCR)

All m^6^A sites on *Atg12* were predicted using SRAMP (http://www.cuilab.cn/sramp). Specific primers targeting high-confidence m^6^A sites were designed for MeRIP-qPCR. Three high-confidence m^6^A sites at positions 285, 531, and 1247 (numbered according to the coding sequence) were selected for validation by MeRIP-qPCR. The primer sequences (5*'*–3*'*) are as follows: *Atg12*-285-F, GAAATGGGCTGTGGAGCGAA; *Atg12*-285-R, GTTCCGAGGCCACCAGTTTA; *Atg12*-531-F, TCAGAGACATTTCGGTGCTGT; *Atg12*-531-R, ACAGGTCCTAACAGATTCATGCT; *Atg12*-1247-F, CCATACCTGCTGTCTCAGCG; *Atg12*-1247-R, GCAGTGCAGTCTAGTTGTGGA. MeRIP-qPCR was performed using the EpiQuik m^6^A RNA Enrichment Kit (Cat. #P-9018-24, Epigentek, Farmingdale, NY, USA). Total RNA was extracted from GC-2 cells using RNA Isolater Total RNA Extraction Reagent. RNA (2 µg) was incubated with m^6^A beads (5 µg) at 4 ℃ with rotation for 1.5 h. The abundance of m^6^A modification was quantified by RT-qPCR, and m^6^A enrichment was calculated using normalized input. Immunoglobulin G (IgG) served as a negative control. Primers were synthesized by Generay (Shanghai, China).

### Chromatin immunoprecipitation-qPCR (ChIP-qPCR)

The ChIP assay was performed using the Magna ChIP A/G kit (Cat. #Magna0017, Millipore, Billerica, MA, USA), according to the manufacturer's protocols. GC-2 cells, at a density of 1 × 10^7^ cells, were treated with 30 μmol/L BDE47 for 24 h and subsequently cross-linked with 1% formaldehyde at room temperature for 10 min. The cross-linking reaction was halted by the addition of 10× glycine and incubation for 5 min. Cells were harvested from each dish using a sterile cell scraper, and the collected cells were centrifuged at 800 *g* for 5 min at 4 ℃ to pellet them. The cell pellet was then resuspended in nuclear lysis buffer. To shear the chromatin into fragments of optimal size, the lysate underwent sonication with an ultrasonic disruptor (Covaris E220, USA), resulting in DNA fragments ranging from 250 to 1000 bp. For immunoprecipitation, an anti-HIF-1α antibody was used and incubated overnight at 4 ℃, with IgG serving as the negative control and RNA polymerase Ⅱ (Pol Ⅱ) serving as a general ChIP procedural control. The enriched DNA was quantified by qPCR. Primers were synthesized by Generay (5′–3′): *Alkbh5*-F, GAGAGCTTTAAAGTGCGGGC; *Alkbh5*-R, GGACGTCATGGACTTGAGCTT; *Atg12*-F, TCCTTAAACTGGTGGCCTCG; *Atg12*-R, CTCTTCCCACAGCACCGAAA.

### Statistical analysis

All data are presented as mean ± SD. Before applying parametric tests, the assumptions of normality and homogeneity of variances were assessed. Normality was checked using the Shapiro-Wilk test, and homogeneity of variances was evaluated using Levene's test. Data were analyzed using parametric tests where their assumptions were considered appropriate (all *P* > 0.05), and Levene's test indicated homogeneity of variances across groups (all *P* > 0.05). Comparisons between two groups were performed using Student's *t-*test, and comparisons among multiple groups were performed using one-way or two-way analysis of variance (ANOVA) followed by Tukey's post hoc test. Statistical analyses were conducted using GraphPad Prism 10 (GraphPad Software Inc., San Diego, CA, USA). All tests were two-tailed, and a *P* value < 0.05 was considered statistically significant.

## Results

### BDE47 induced CYP3A11 expression and ferroptosis in mouse testes

**Figure 1:**
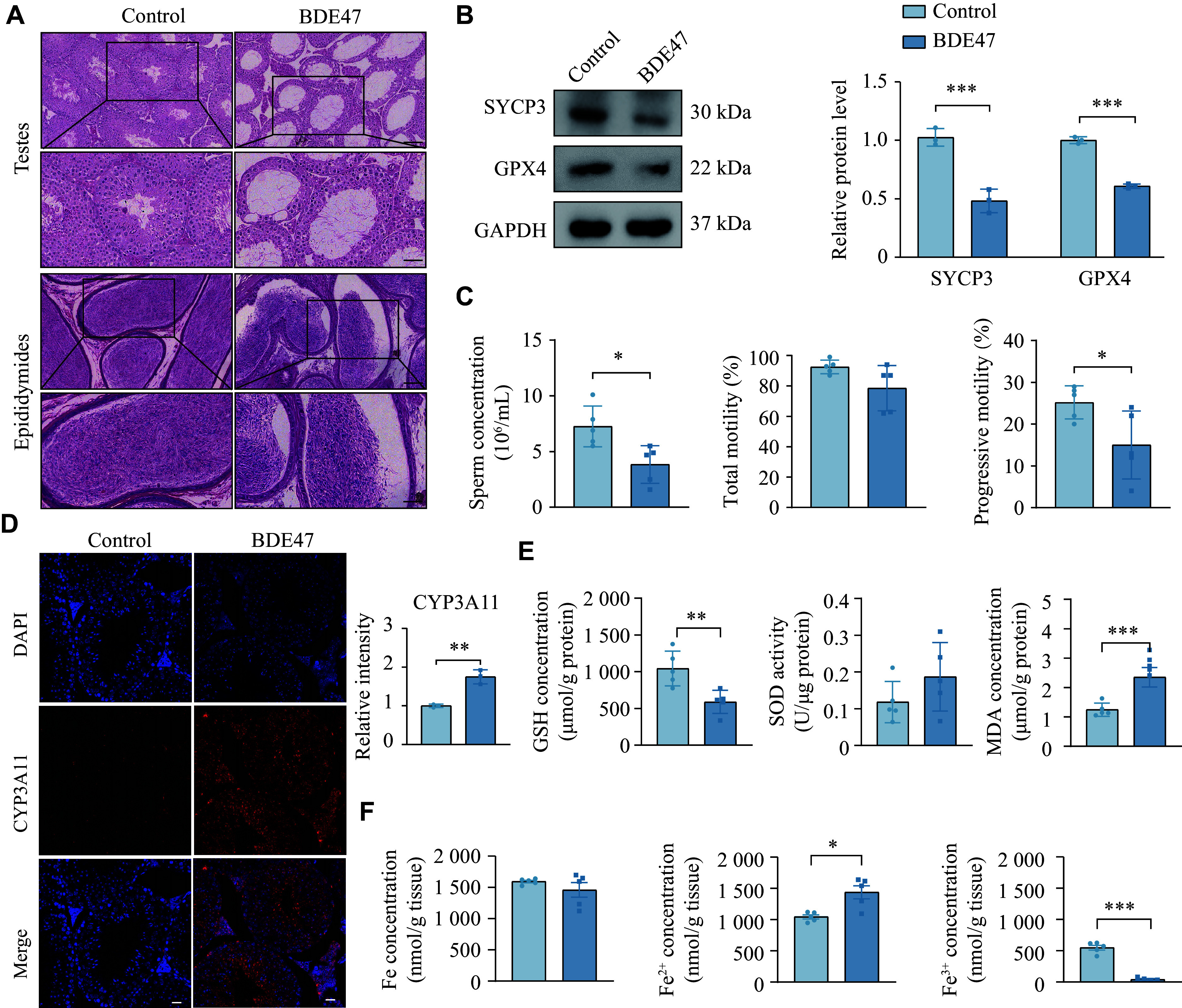
Ferroptosis in BDE47-treated male mice. Male C57BL/6 mice (*n* = 5 per group) received BDE47 (1.5 mg/kg) by oral gavage for 8 weeks. The control group received saline with an equal amount of DMSO. A: Histological observations of the seminiferous tubules and epididymis were performed using hematoxylin and eosin staining. Representative overview (top) and magnified images (bottom) are shown. Scale bars: 50 μm and 100 μm, respectively. B: SYCP3 and GPX4 expression in mouse testes was analyzed by Western blotting, with GAPDH as a loading control. C: Epididymal sperm quality was assessed using computer-assisted sperm analysis. Parameters included sperm concentration, total motility, and progressive motility. D: CYP3A11 expression in mouse testes was detected by immunofluorescence staining. DAPI stained nuclei (blue), and Alexa Fluor 594 labeled CYP3A11 (red). Representative images and quantitative analysis are shown. Scale bar: 20 μm. E: Testicular glutathione (GSH), superoxide dismutase (SOD), and malondialdehyde (MDA) levels were measured by colorimetric assays. F: The levels of total iron (Fe), ferrous (Fe^2+^), and ferric iron (Fe^3+^) in testes were measured using colorimetric iron assay kits. Fluorescence intensity in panel D was quantified using ImageJ software, and the fluorescence intensity from three independent samples was analyzed. Data are presented as mean ± standard deviation. Statistical analysis was performed using Student's *t*-test with ^*^P < 0.05, ^**^P < 0.01, and ^***^P < 0.001. Abbreviations: BDE47, 2,2',4,4'-tetrabromodiphenyl ether; CYP3A11, cytochrome P450 family 3 subfamily A member 11; GPX4, glutathione peroxidase 4; SYCP3, synaptonemal complex protein 3.

BDE47, a CYP3A metabolic substrate, is linked to reproductive toxicity. After oral administration of 1.5 mg/kg BDE47 to C57BL/6 mice for 8 weeks, histological examination revealed disorganized seminiferous tubules, sloughing of the spermatogenic epithelium, and reduced epididymal sperm density in BDE47-treated mice (***Fig. 1A***). Western blotting analysis showed that BDE47 significantly decreased the expression of the ferroptosis marker GPX4 and the spermatocyte marker SYCP3 (***Fig. 1B***). Computer-assisted sperm analysis further demonstrated a significant reduction in total sperm concentration and progressive sperm percentage in the BDE47-treated group (***Fig. 1C***). These results showed that BDE47 caused damage to germ cells. Immunostaining analysis demonstrated that BDE47 specifically induced CYP3A11 expression in the testes, indicating that BDE47 upregulates its own metabolic enzyme in this tissue (***Fig. 1D***). Further analysis revealed that BDE47 significantly depleted testicular GSH levels and elevated MDA levels (***Fig. 1E***). In addition, BDE47 increased Fe^2+^ levels in the testes of mice (***Fig. 1F***). These results collectively indicate that BDE47 exposure induces ferroptosis-associated changes in the testes. Despite the high baseline expression of CYP3A in the liver, BDE47 did not induce significant hepatic ferroptosis or further CYP3A upregulation (***Supplementary Fig. 1***), suggesting that, under the exposure conditions used in this study, prominent ferroptosis-associated changes were observed in the testes but not in the liver.

### BDE47 induced ferroptosis in GC-2 cells

**Figure 2:**
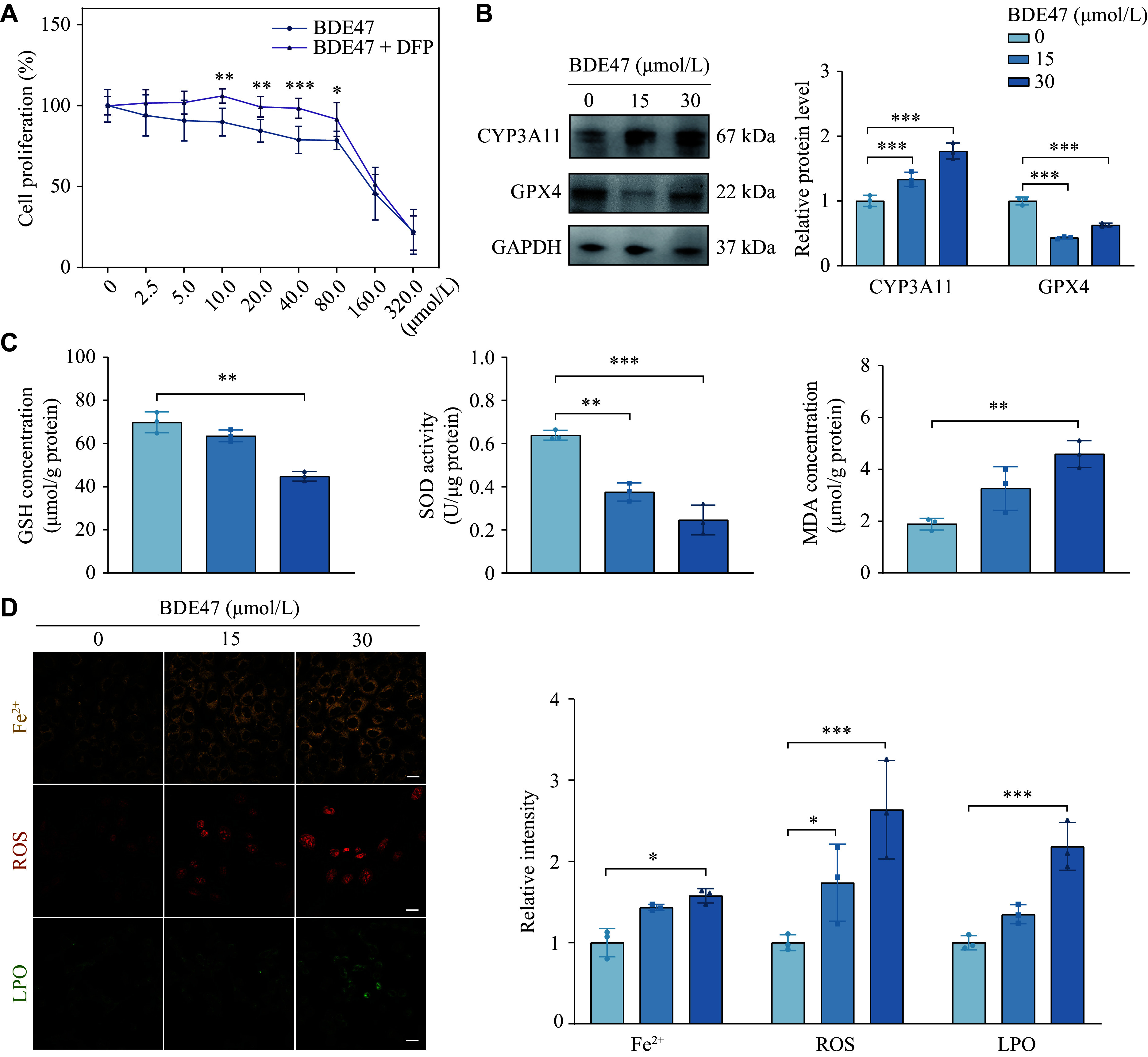
Ferroptosis in BDE47-treated GC-2 cells. For the CCK-8 assay, cells were treated with BDE47 alone (0–320 μmol/L) or in combination with 1 μmol/L deferiprone (DFP) for 48 h. Each group had six replicates per experiment; for other experiments, GC-2 cells received BDE47 (0, 15, 30 μmol/L) for 48 h (*n* = 3 per group). A: Cytotoxicity of BDE47 and the effect of DFP (1 μmol/L) in GC-2 cells were evaluated by the CCK-8 assay. B: Expression of GPX4 and CYP3A11 in GC-2 cells was analyzed by Western blotting, and quantitative analysis was performed, with GAPDH as a loading control. C: Glutathione (GSH), superoxide dismutase (SOD), and malondialdehyde (MDA) levels in GC-2 cells were measured by colorimetric assays. D: The levels of reactive oxygen species (ROS), Fe^2+^, and lipid peroxide (LPO) in GC-2 cells were measured using fluorescence probes DHE, FerroOrange, and Liperfluo, respectively. Representative fluorescence images and quantitative analysis are shown. Scale bar: 20 μm. Fluorescence intensity was measured using ImageJ software. Data are presented as mean ± standard deviation. Statistical analysis was performed using one-way ANOVA with Tukey's post hoc test. ^*^P < 0.05, ^**^P < 0.01, and ^***^P < 0.001. Abbreviations: BDE47, 2,2',4,4'-tetrabromodiphenyl ether; CYP3A11, cytochrome P450 family 3 subfamily A member 11; GPX4, glutathione peroxidase 4.

To further verify BDE47-induced ferroptosis, we treated GC-2 cells with BDE47. The cell viability assay revealed that 80 μmol/L BDE47 induced approximately 20% cell viability loss, which was partially rescued by the free iron chelating agent DFP (***Fig. 2A***). Based on these results, we selected 0, 15, and 30 μmol/L BDE47 for subsequent experiments. Western blotting analysis showed a decrease in GPX4 expression and an increase in CYP3A11 expression in GC-2 cells treated with 30 μmol/L BDE47 (***Fig. 2B***). Oxidative stress profiles revealed significant GSH depletion and increased MDA levels in BDE47-treated cells (***Fig. 2C***). Consistently, BDE47 treatment induced a clear dose-dependent accumulation of ferroptotic markers in GC-2 cells, including elevated Fe^2+^, increased ROS, and enhanced LPO levels (***Fig. 2D***). These findings support the induction of a ferroptosis-like, iron-dependent lipid-peroxidative phenotype by BDE47 in GC-2 cells.

### BDE47 induced ferritinophagy in mice and GC-2 cells

**Figure 3:**
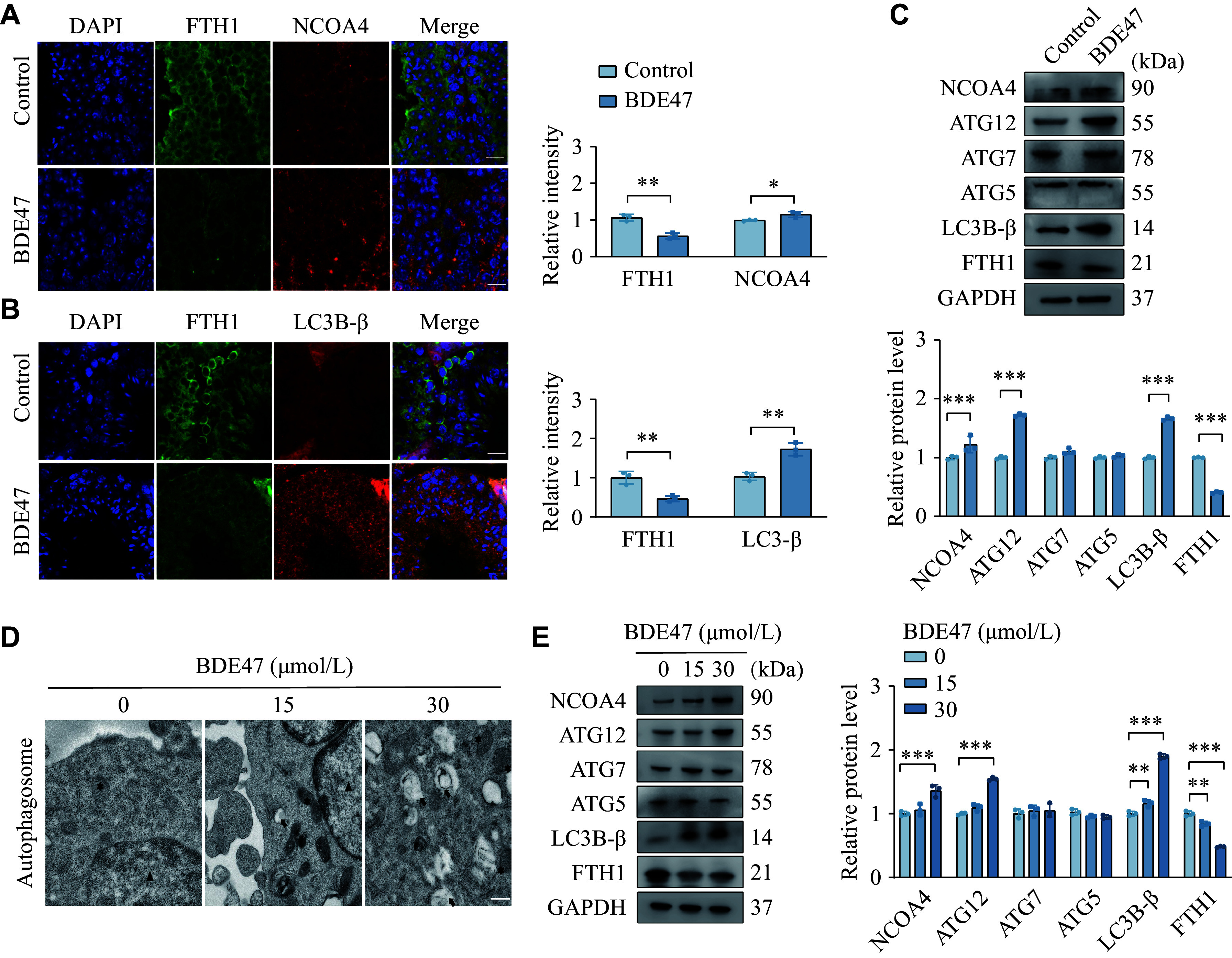
BDE47 induced ferritinophagy in mice and GC-2 cells. Male C57BL/6 mice (*n* = 5 per group) received BDE47 (1.5 mg/kg) by oral gavage for 8 weeks. The control group received saline with an equal amount of DMSO. GC-2 cells received BDE47 (0, 15, 30 μmol/L) for 48 h (*n* = 3 per group). A: Immunofluorescence staining was performed to detect FTH1 and NCOA4 expression in mouse testes. DAPI stained nuclei (blue), green fluorescence indicates FTH1 (520 nm), and red fluorescence indicates NCOA4 (570 nm). Representative images and quantitative analysis are shown. Scale bar: 20 μm. B: Immunofluorescence staining was performed to detect FTH1 and LC3-β expression in mouse testes. DAPI stained nuclei (blue), green fluorescence indicates FTH1 (520 nm), and red fluorescence indicates LC3-β (570 nm). Representative images and quantitative analysis are shown. Scale bar: 20 μm. C: Western blotting analyses of FTH1, NCOA4, and autophagy-related protein expression in testes, with GAPDH as a loading control. D: Representative images of autophagosomes in GC-2 cells treated with BDE47 were observed using electron microscopy. Black arrows, autophagosomes; asterisks, mitochondria; triangles, cell nuclei. Scale bar: 500 nm. E: Western blotting analyses of FTH1, NCOA4, and autophagy-related protein expression in GC-2 cells, with GAPDH as a loading control. Quantitative analyses are shown. Fluorescence intensity was measured using ImageJ software. Data are presented as mean ± standard deviation. Statistical analysis was performed using Student's *t*-test (A, B, and C) or one-way ANOVA with Tukey's post hoc test (E). ^*^P < 0.05, ^**^P < 0.01, and ^***^P < 0.001. Abbreviations: ATG5, autophagy-related 5; ATG7, autophagy-related 7; ATG12, autophagy-related 12; BDE47, 2,2',4,4'-tetrabromodiphenyl ether; FTH1, ferritin heavy chain 1; LC3-β, microtubule associated protein 1 light chain 3 beta; NCOA4, nuclear receptor coactivator 4.

Ferritinophagy is an important regulatory pathway that mediates ferroptosis. We therefore hypothesized that BDE47 may cause ferroptosis through ferritinophagy. Immunofluorescence staining revealed decreased FTH1 expression and a slight increase in NCOA4 in the testes of BDE47-exposed mice (***Fig. 3A***). The autophagy marker microtubule-associated protein 1 light chain 3 beta (LC3-β) was increased in the testes and colocalized with FTH1 (***Fig. 3B***). Electron microscopy of BDE47-treated GC-2 cells revealed the formation of autophagic vesicles, with pronounced accumulation at the higher dose (30 μmol/L) (***Fig. 3D***). These results suggest that the accumulation of autophagy-associated structures and proteins may contribute to the downregulation of FTH1. Western blotting analysis further validated the expression changes of FTH1, NCOA4, and autophagic proteins. In addition to causing a significant decrease in FTH1 and a slight increase in NCOA4, BDE47 significantly upregulated autophagy-related 12 (ATG12) in both testicular tissue and GC-2 cells (***Fig. 3C*** and ***3E***).

### ATG12-mediated autophagy contributed to BDE47-induced ferritinophagy

**Figure 4:**
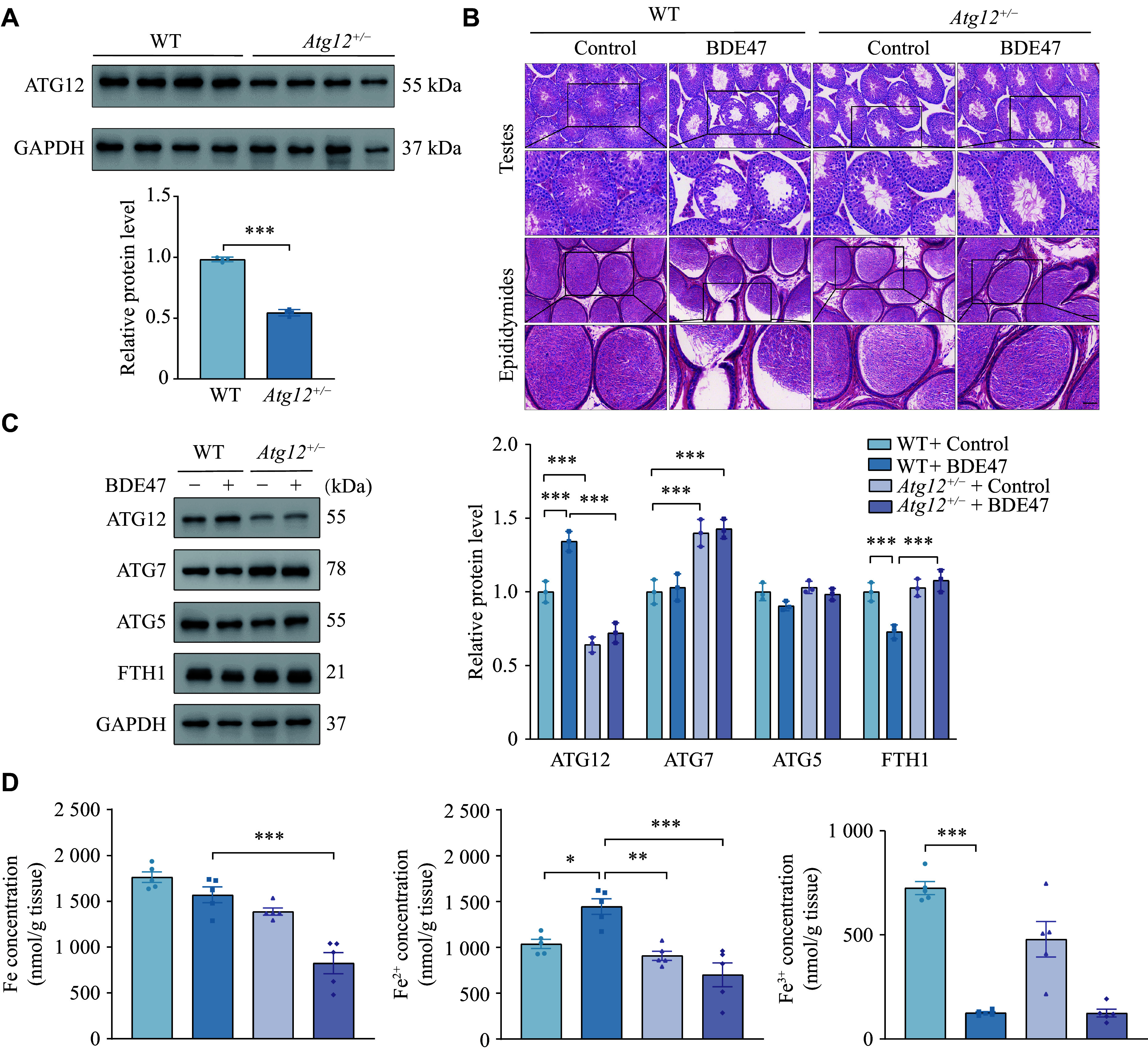
ATG12-mediated autophagy contributed to BDE47-induced ferritinophagy in mice. A: ATG12 expression in wild-type (WT) and *Atg12*^*+/*−^ mice was detected using Western blotting (*n* = 4 per group). GAPDH served as a loading control. Band intensities were quantified by ImageJ software. B: Histopathological observation of seminiferous tubules from WT and *Atg12*^*+/*−^ mice treated with BDE47 (1.5 mg/kg) or saline with an equal amount of DMSO (control) for 8 weeks (*n* = 5 per group). Representative images with magnified views of the luminal architecture are shown. Scale bars: 50 μm and 100 μm, respectively. C: Expression of autophagy-related proteins and FTH1 in testes of WT and *Atg12*^*+/*−^ mice was detected using Western blotting. GAPDH served as a loading control. D: Iron accumulation (ferrous, ferric, and total iron) in testes of BDE47-treated WT and *Atg12*^*+/*−^ mice was measured by colorimetric assay kits. Data are presented as mean ± standard deviation. Statistical analysis was performed using Student's *t*-test (A) or two-way ANOVA with Tukey's post hoc test (C and D). ^*^P < 0.05, ^**^P < 0.01, and ^***^P < 0.001. Abbreviations: ATG5, autophagy-related 5; ATG7, autophagy-related 7; ATG12, autophagy-related 12; BDE47, 2,2',4,4'-tetrabromodiphenyl ether; FTH1, ferritin heavy chain 1.

Because complete knockout of *Atg12* causes embryonic lethality in mice, we employed an *Atg12* heterozygous knockdown mouse model (*Atg12*^+/−^) to investigate the role of ATG12 in the autophagic process. As shown in ***Fig. 4A***, *Atg12*^+/−^ mice showed an approximately 50% reduction in ATG12 expression compared with wild-type controls. Using this model, we further investigated the role of ATG12 in ferritinophagy induced by the CYP3A substrate BDE47. H&E staining of mouse testes and epididymides revealed that *Atg12* haploinsufficiency attenuated BDE47-induced testicular damage (***Fig. 4B***). Furthermore, *Atg12* haploinsufficiency significantly attenuated the BDE47-induced upregulation of ATG12 and downregulation of FTH1 in *Atg12*^+/−^ mice (***Fig. 4C***). We also measured tissue iron content and found that Fe^2+^ levels were significantly lower in BDE47-treated *Atg12*^+/−^ mice than in BDE47-treated wild-type mice (***Fig. 4D***). These results support a role for ATG12-dependent autophagy in BDE47-associated FTH1 loss and iron accumulation, suggesting that ATG12 is involved in ferritinophagy.

### BDE47 induced ATG12 upregulation through the m^6^A modification pathway

**Figure 5:**
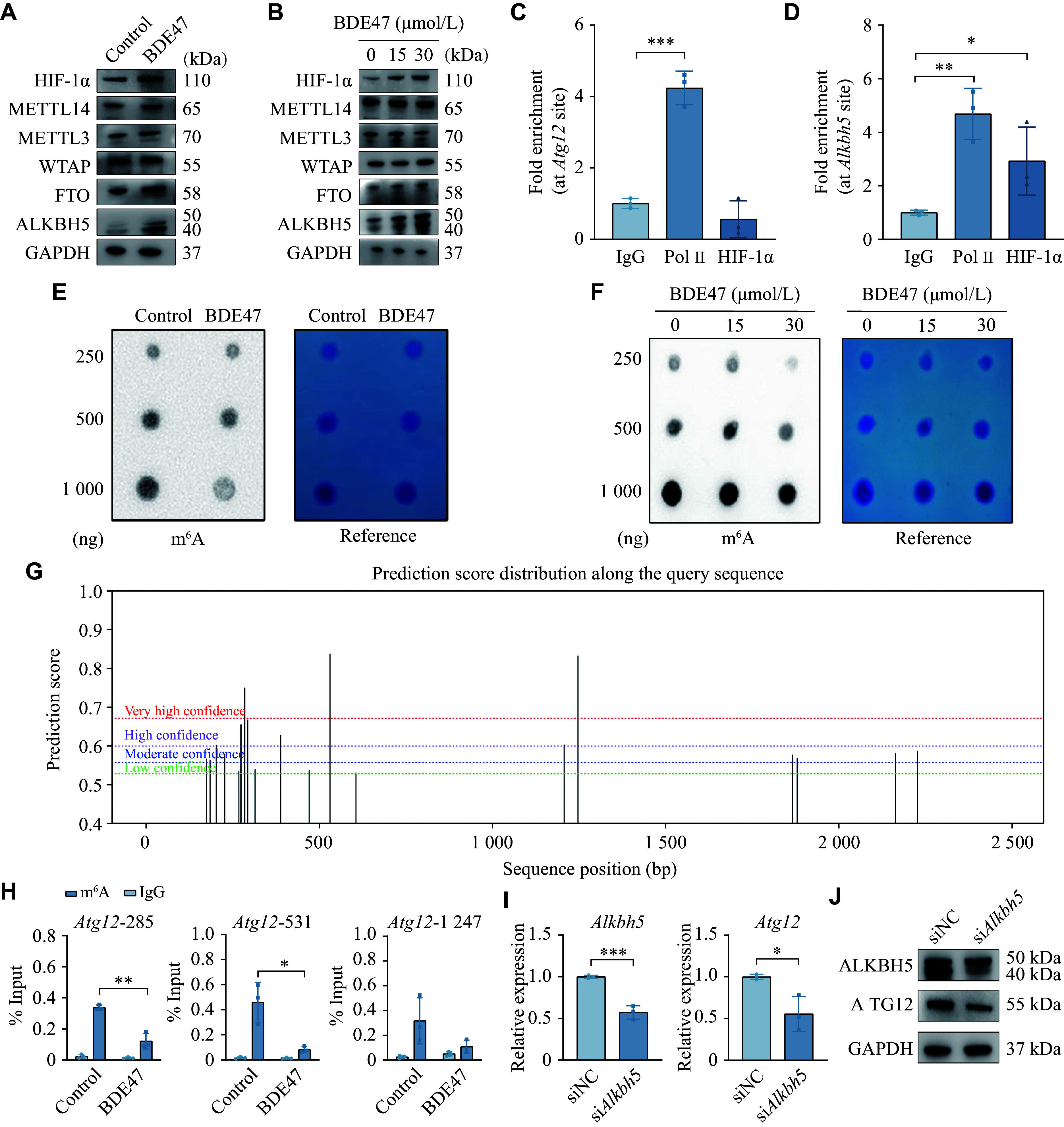
m^6^A modification and related regulatory pathway in testes and GC-2 cells treated with BDE47. Male C57BL/6 mice (*n* = 5 per group) received BDE47 (1.5 mg/kg) by oral gavage for 8 weeks; the control group received saline with an equal amount of DMSO. GC-2 cells received BDE47 (0, 15, 30 μmol/L) for 48 h (*n* = 3 per group). A and B: Expression of HIF-1α and m^6^A-related regulators (METTL14, METTL3, WTAP, FTO, ALKBH5) in testicular tissues of mice (A) and GC-2 cells (B) detected using Western blotting analyses. GAPDH was used as a loading control. Quantification analyses are shown in ***Supplementary Fig. 2***. C and D: ChIP-qPCR analysis was performed to detect HIF-1α binding to the promoter regions of *Atg12* (C) and *Alkbh5* (D) in GC-2 cells. IgG and RNA polymerase Ⅱ (Pol Ⅱ) served as a negative control and a general ChIP procedural control, respectively. Data were normalized to input DNA. E and F: Total m^6^A levels in testicular RNA of mice and GC-2 cells were detected by dot blotting analyses. Methylene blue staining served as a reference for RNA loading. G: Prediction of m^6^A methylation sites in *Atg12* mRNA using SRAMP (http://www.cuilab.cn/sramp). High-confidence m^6^A peaks are shown at nucleotide positions 285, 531, and 1247. H: m^6^A enrichment at *Atg12* sites (285, 531, and 1247) in GC-2 cells treated with BDE47 was detected using the MeRIP-qPCR assay. Results were normalized to input RNA. I and J: Expression of *Alkbh5* mRNA (I) and ATG12 protein (J) in GC-2 cells after *Alkbh5* siRNA interference was detected by RT-qPCR and Western blotting, respectively. GAPDH served as a loading control. Quantification analyses are shown in ***Supplementary Fig. 2***. Data are presented as mean ± standard deviation. Statistical analysis was performed using Student's *t*-test (A, C, D, H, I, and J) or one-way ANOVA with Tukey's post hoc test (B). ^*^P < 0.05, ^**^P < 0.01, and ^***^P < 0.001. Abbreviations: ALKBH5, AlkB homolog 5; BDE47, 2,2',4,4'-tetrabromodiphenyl ether; FTO, fat mass and obesity-associated protein; HIF-1α, hypoxia-inducible factor 1α; m^6^A, N6-methyladenosine; METTL3, methyltransferase-like 3; METTL14, methyltransferase-like 14; WTAP, Wilms tumor 1-associating protein.

To elucidate how oxidative stress enhances autophagy and promotes ferritin degradation, we first examined the role of hypoxia-inducible factor 1α (HIF-1α), a key transcriptional regulator activated under oxidative stress. Western blotting analysis showed that BDE47 significantly upregulated HIF-1α expression in both testicular tissues and GC-2 cells (***Fig. 5A*** and ***5B***; ***Supplementary Fig. 2A*** and ***2B***). However, ChIP-qPCR revealed that HIF-1α did not directly bind to the *Atg12* promoter (***Fig. 5C***). Since HIF-1α is known to transcriptionally regulate *Alkbh5*^[25]^, an m^6^A demethylase, we hypothesized that BDE47 might modulate ATG12 expression through m^6^A-dependent post-transcriptional regulation. ChIP-qPCR further demonstrated that HIF-1α directly binds to the *Alkbh5* promoter (***Fig. 5D***). Dot blotting analysis demonstrated a global reduction in m^6^A methylation levels in BDE47-treated testicular tissues and in GC-2 cells treated with 30 μmol/L BDE47 (***Fig. 5E*** and ***5F***). Additionally, we observed a significant increase in ALKBH5 protein levels in both BDE47-treated testicular tissues and 30 μmol/L BDE47-treated GC-2 cells (***Fig. 5A*** and ***5B***). These results suggest that ALKBH5 may play a key role in the regulation of ATG12.

To pinpoint the specific m^6^A modification sites on *Atg12* mRNA, we performed a bioinformatic prediction that identified sites 285, 531, and 1247 as the regions most likely to have high m^6^A abundance (***Fig. 5G***). BDE47 exposure reduced m^6^A modification at sites 285 and 531 (***Fig. 5H***). Furthermore, *Alkbh5* knockdown in GC-2 cells led to downregulation of *Atg12* mRNA and ATG12 protein levels, suggesting a regulatory role of ALKBH5 in ATG12 expression (***Fig. 5I*** and ***5J***; ***Supplementary Fig. 2C***).

### NAC and DFP reversed BDE47-induced ferritinophagy

**Figure 6:**
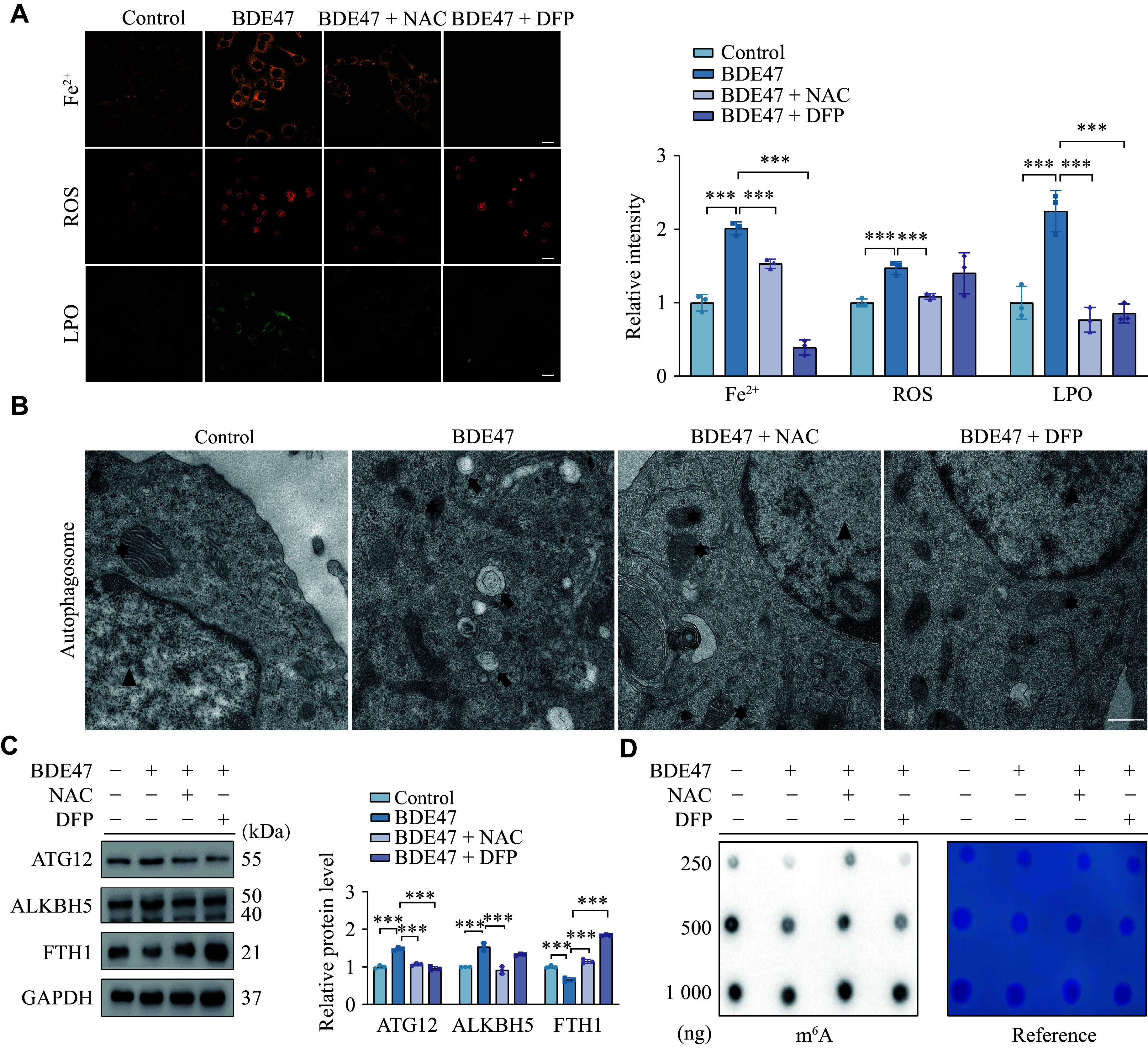
N-Acetylcysteine (NAC) and deferiprone (DFP) attenuated ferritinophagy of GC-2 cells treated with BDE47. GC-2 cells were treated with BDE47 (30 μmol/L) alone or combined with NAC (5 mmol/L) or DFP (1 μmol/L) for 48 h (*n* = 3 per group). A: Oxidative stress and ferroptosis in GC-2 cells were detected using fluorescence probe assays. Representative fluorescence images and quantitative analysis are shown. Scale bar: 20 μm. B: Autophagosome formation in GC-2 cells was detected by transmission electron microscopy. Black arrows, autophagosomes; asterisks, mitochondria; triangles, nuclei. Scale bar: 500 nm. C: Expression of ATG12, ALKBH5, and FTH1 in GC-2 cells was analyzed by Western blotting, and quantitative analysis was performed. GAPDH served as a loading control. D: Total RNA m^6^A levels in GC-2 cells were detected using dot blot analysis. Methylene blue staining served as an internal reference. Fluorescence intensity in panel A was measured using ImageJ software. Data are presented as mean ± standard deviation. Statistical analysis was performed using one-way ANOVA with Tukey's post hoc test. ^***^P < 0.001. Abbreviations: ALKBH5, AlkB homolog 5; ATG12, autophagy-related 12; BDE47, 2,2',4,4'-tetrabromodiphenyl ether; FTH1, ferritin heavy chain 1; m^6^A, N6-methyladenosine.

To further investigate the role of ROS in regulating ferritinophagy, we employed a pharmacological intervention strategy. We treated GC-2 cells with 30 μmol/L BDE47 alone or in combination with either NAC (a typical antioxidant) or DFP (a free iron chelating agent). NAC and DFP both reduced BDE47-induced LPO levels. However, they acted through distinct mechanisms: NAC scavenged ROS as an antioxidant, whereas DFP chelated iron and completely prevented the BDE47-induced increase in Fe^2+^ (***Fig. 6A***). NAC and DFP also reduced the formation of autophagic vesicles triggered by BDE47 (***Fig. 6B***). Moreover, NAC and DFP both reversed the BDE47-induced upregulation of ATG12, as well as the downregulation of FTH1 (***Fig. 6C***). NAC effectively reversed the BDE47-induced upregulation of ALKBH5 expression and the reduction in m^6^A methylation levels (***Fig. 6C*** and ***6D***). These results suggest that ROS may play an important role in ferritinophagy induced by BDE47.

### Hydrogen peroxide reproduced ferritinophagy-associated molecular changes

**Figure 7:**
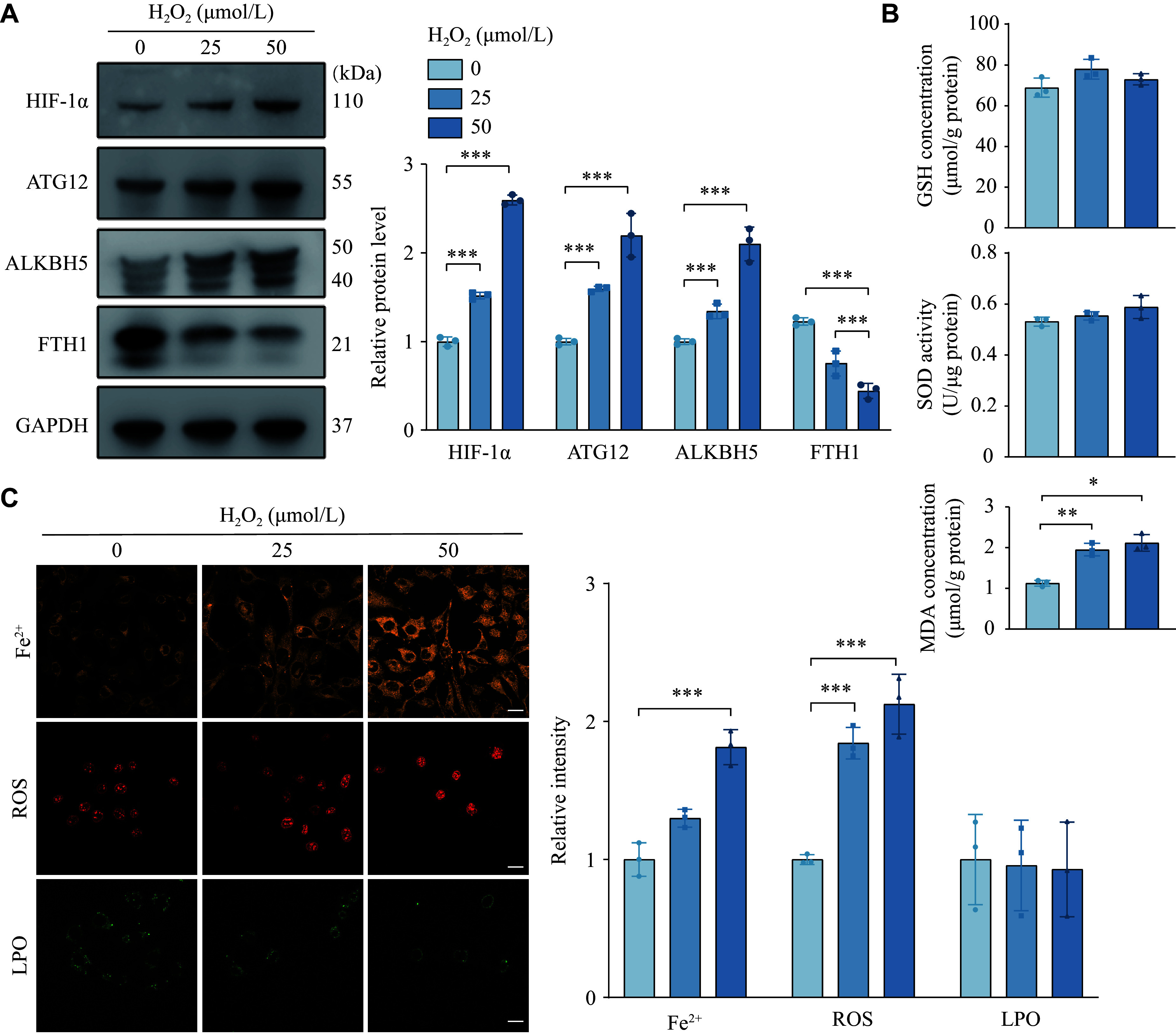
H_2_O_2_ induced ferritinophagy in GC-2 cells. GC-2 cells were treated with H_2_O_2_ (0, 25, and 50 μmol/L) for 48 h (*n* = 3 per group). A: Expression of HIF-1α, ATG12, ALKBH5, and FTH1 in GC-2 cells treated with H_2_O_2_ was analyzed by Western blotting, and quantitative analysis was performed. GAPDH was used as a loading control. B: Oxidative stress markers (GSH, SOD, and MDA) in GC-2 cells treated with H_2_O_2_ were measured by colorimetric assays. C: Levels of ROS (DHE), Fe^2+^ (FerroOrange), and LPO (Liperfluo) in GC-2 cells treated with H_2_O_2_ were detected using fluorescence probe assays. Representative fluorescence images and quantitative analysis are shown. Scale bar: 20 μm. Fluorescence intensity was measured using ImageJ software. Data are presented as mean ± standard deviation. Statistical analysis was performed using one-way ANOVA with Tukey's post hoc test. ^*^P < 0.05, ^**^P < 0.01, and ^***^P < 0.001. Abbreviations: ALKBH5, AlkB homolog 5; ATG12, autophagy-related 12; FTH1, ferritin heavy chain 1; GSH, glutathione; HIF-1α, hypoxia-inducible factor 1α; MDA, malondialdehyde; SOD, superoxide dismutase.

To directly test the role of ROS, we treated GC-2 cells with H_2_O_2_, a typical reactive oxygen species. Our results showed that 25 μmol/L and 50 μmol/L H_2_O_2_ not only initiated iron-associated ferritinophagy but also upregulated ATG12 expression (***Fig. 7A***). H_2_O_2_ exposure also decreased FTH1 expression (***Fig. 7A***). Furthermore, H_2_O_2_-induced oxidative stress caused significant Fe^2^^+^ accumulation and elevated MDA levels (***Fig. 7B*** and ***7C***). Since ROS are important byproducts of CYP3A-mediated BDE47 metabolism, these findings further suggest a regulatory role for ROS in downstream ferritinophagy.

### CYP3A4 overexpression potentiated BDE47-induced ferroptosis

**Figure 8:**
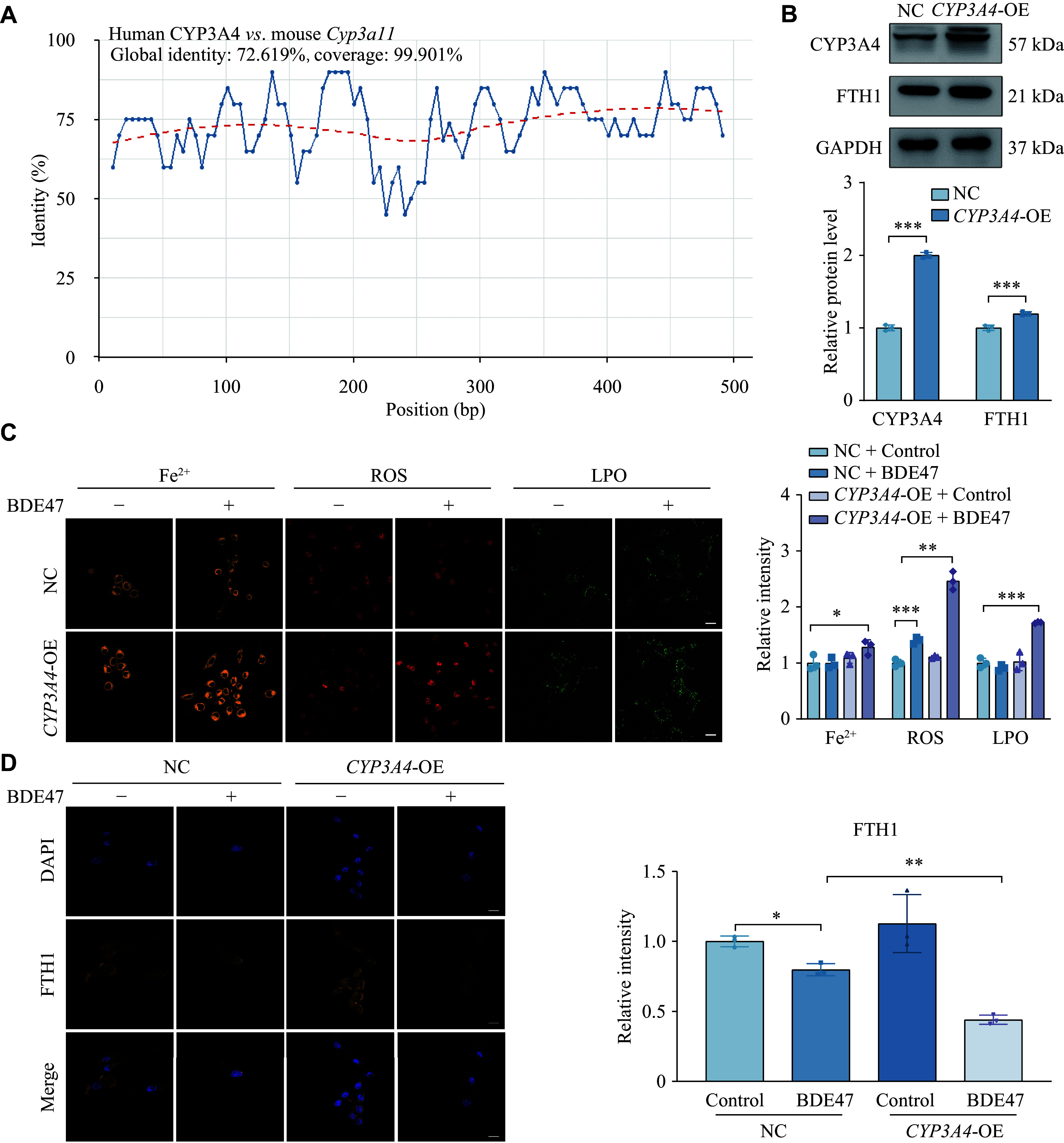
Effects of CYP3A4 overexpression on ferritinophagy in GC-2 cells treated with BDE47. A: Amino acid sequence alignment of human *CYP3A4* and mouse *Cyp3a11* was performed. The X-axis represents amino acid position, and the Y-axis indicates sequence identity percentage. Blue line, sliding identity; red dashed line, global identity. B: Expression of CYP3A4 and FTH1 in GC-2 cells overexpressing CYP3A4 (*CYP3A4*-OE) was analyzed by Western blotting, and quantitative analysis was performed. The GC-2 cells with a vehicle plasmid served as a negative control (NC). GAPDH served as a loading control. C: Oxidative stress levels in NC and *CYP3A4*-OE GC-2 cells following BDE47 treatment (15 μmol/L, 48 h) were detected by fluorescence probe assays. DHE for ROS, FerroOrange for Fe^2+^, and Liperfluo for LPO. Representative fluorescence images and quantitative analysis are shown. Scale bar: 20 μm. D: FTH1 expression in NC and *CYP3A4*-OE GC-2 cells after treatment with BDE47 (15 μmol/L, 48 h) was detected by using an immunofluorescence assay. DAPI was used to stain nuclei. Representative images and quantitative analysis were obtained. Scale bar: 20 μm. Fluorescence intensity was measured using ImageJ software. Data are presented as mean ± standard deviation. Statistical analysis was performed using two-way ANOVA with Tukey's post hoc test. ^*^P < 0.05, ^**^P < 0.01, and ^***^P < 0.001. Abbreviations: BDE47, 2,2',4,4'-tetrabromodiphenyl ether; CYP3A4, cytochrome P450 3A4; FTH1, ferritin heavy chain 1.

Mouse *Cyp3a11* shares high homology with human *CYP3A4*, showing substantial sequence conservation, although species-specific differences in substrate metabolism cannot be excluded (global identity = 72.6%, coverage = 99.9%) (***Fig. 8A***). Lentivirus-mediated overexpression of CYP3A4 (*CYP3A4*-OE) exacerbated ferroptotic susceptibility: upon treatment with 15 μmol/L BDE47, cells with *CYP3A4*-OE exhibited higher ROS, Fe^2+^, and LPO levels than controls (***Fig. 8B*** and ***8C***). Notably, CYP3A4 overexpression also significantly aggravated the BDE47-induced reduction in FTH1 levels (***Fig. 8D***), further highlighting the pivotal role of CYP3A-mediated metabolism in xenobiotic-induced ferroptosis.

### *Cyp3a11* knockdown attenuated BDE47-induced oxidative stress and ferritinophagy in GC-2 cells

**Figure 9:**
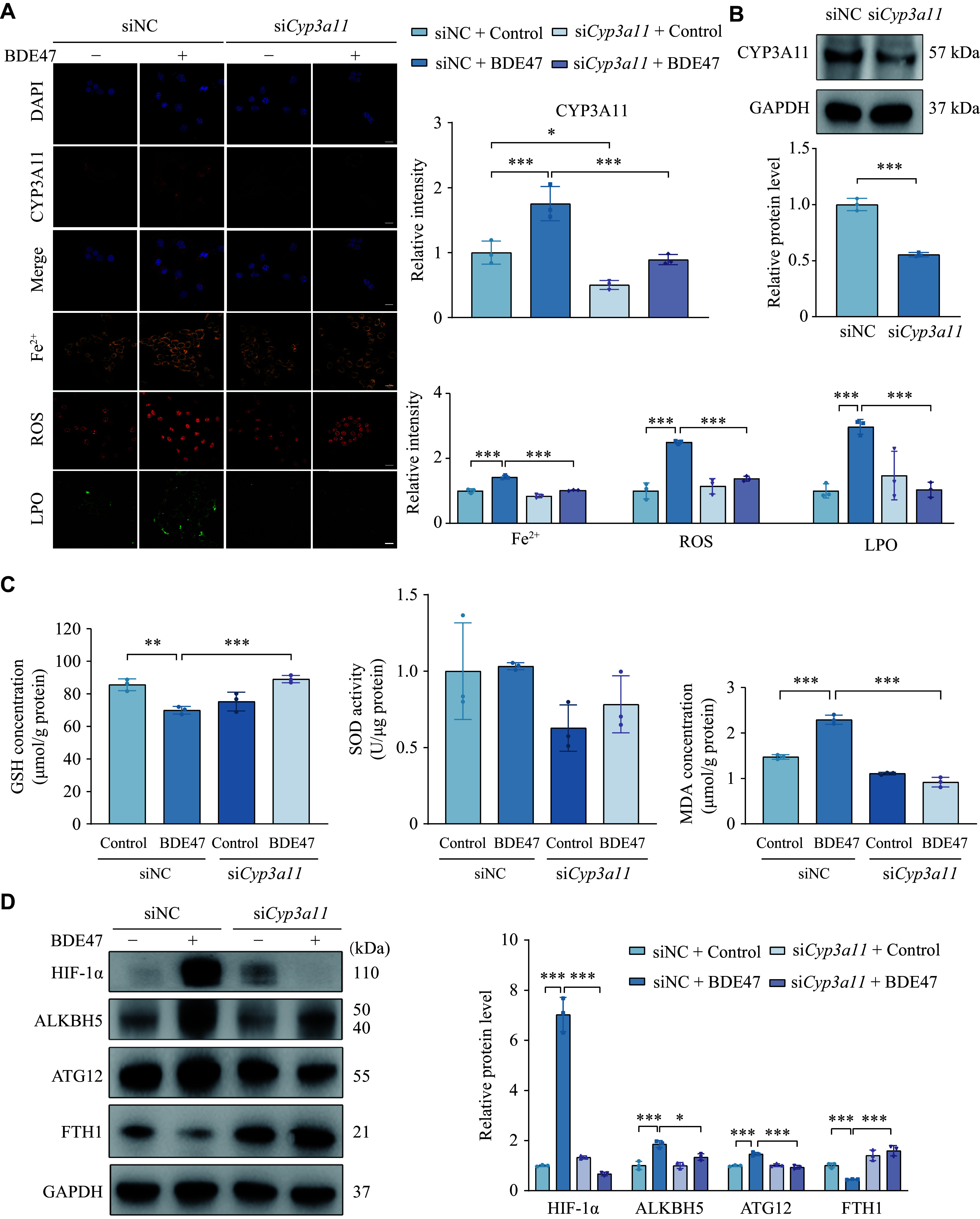
Knockdown of *Cyp3a11* in GC-2 cells rescues BDE47-induced ferritinophagy. GC-2 cells were transfected with siRNA targeting *Cyp3a11* (si*Cyp3a11*) or negative control siRNA (siNC) and treated with BDE47 (30 μmol/L, 48 h). A: CYP3A11 expression in cells was detected by immunofluorescence staining, and levels of ROS, Fe^2+^, and LPO were measured using fluorescence probe assays. Fluorescence probes: DHE for reactive oxygen species (ROS), FerroOrange for Fe^2+^, and Liperfluo for lipid peroxide (LPO). Representative fluorescence images and quantitative analysis are shown. Scale bar: 20 μm. B: Knockdown efficiency of CYP3A11 in GC-2 cells was validated by Western blotting. GAPDH served as an internal loading control. C: The levels of glutathione (GSH), superoxide dismutase (SOD), and malondialdehyde (MDA) in siNC or si*Cyp3a11* cells were determined. D: Autophagy- and ferritinophagy-related proteins (HIF-1α, ALKBH5, ATG12, and FTH1) in siNC and si*Cyp3a11* cells were detected using Western blotting. GAPDH served as a loading control. Fluorescence intensity was measured using ImageJ software. Data are presented as mean ± standard deviation. Statistical analysis was performed using two-way ANOVA with Tukey's post hoc test. ^*^P < 0.05, ^**^P < 0.01, and ^***^P < 0.001. Abbreviations: ALKBH5, AlkB homolog 5; ATG12, autophagy-related 12; BDE47, 2,2',4,4'-tetrabromodiphenyl ether; FTH1, ferritin heavy chain 1; HIF-1α, hypoxia-inducible factor 1α; m^6^A, N6-methyladenosine.

To further verify the role of CYP3A11 in mediating oxidative stress and ferritinophagy, we knocked down *Cyp3a11* in GC-2 cells using specific siRNA (si*Cyp3a11*), which reduced CYP3A11 expression by approximately 50% (***Fig. 9A*** and ***9B***). si*Cyp3a11* also significantly suppressed the BDE47-induced upregulation of CYP3A11. Moreover, the elevated ROS, Fe^2+^, and LPO levels caused by BDE47 were significantly reduced by si*Cyp3a11* knockdown (***Fig. 9A***). Further analysis revealed that *Cyp3a11* knockdown reversed both the BDE47-induced depletion of GSH and the increase in MDA (***Fig. 9C***). Additionally, *Cyp3a11* knockdown attenuated the BDE47-induced upregulation of ATG12 and downregulation of FTH1, and reversed the BDE47-mediated induction of HIF-1α and ALKBH5 (***Fig. 9D***).

## Discussion

BDE47 is a known metabolic substrate of CYP3A, which can induce CYP3A expression and be metabolically activated to generate hydroxylated metabolites^[8]^. Previous studies have mainly focused on the toxicity of the generated hydroxylated metabolites. In the current study, however, we focused on the role of CYP3A itself in BDE47-induced reproductive damage. The high expression of CYP3A caused by BDE47 not only increases ROS levels, but may also indirectly cause Fe^2+^ overload^[26]^. In GC-2 cells, both the knockdown and overexpression of CYP3A altered BDE47-induced ferroptosis, supporting that CYP3A upregulation drives ROS and Fe^2+^ accumulation. As the most important phase Ⅰ metabolic enzyme, CYP3A is mainly expressed in the liver to mediate the metabolism of xenobiotics^[27]^. Although BDE47 can cause liver damage at higher doses with long-term exposure^[28]^, no significant liver damage was observed in our study at the administered dose and time point. Instead, testicular damage occurred through the upregulation of CYP3A11 and elevated ROS and Fe^2+^, suggesting that, under the exposure conditions examined, the testes exhibited greater susceptibility than the liver to BDE47-associated CYP3A induction and ferroptosis-related changes. This may be attributed to two factors: (1) the liver has high levels of phase Ⅱ metabolic enzymes that can effectively eliminate toxic products generated by CYP3A metabolism, whereas the testes do not^[29]^; and (2) the complexity of the testicular spermatogenesis process renders it more sensitive to ROS^[17]^.

Among the P450 isoforms examined, CYP3A exhibits the highest activity in catalyzing NADPH oxidation, generating superoxide anion radicals, producing NADPH-dependent chemiluminescence, oxidizing dichlorofluorescein diacetate, and reducing both ferric-EDTA and ferric-citrate^[30]^. The CYP3A catalytic cycle involves dynamic iron redox transitions essential for substrate oxidation. NADPH-cytochrome P450 reductase delivers electrons to the heme iron (Fe^3+^→Fe^2+^), enabling oxygen activation and formation of a high-valent iron-oxo intermediate (FeO^3+^)^[31–32]^. This cycle not only produces substantial fluxes of superoxide (O_2_·^−^) and H_2_O_2_, which disrupt redox homeostasis and protein translation, but may also increase Fe^2+^ levels through ROS-related regulatory pathways. The resultant Fe^2+^ and ROS synergistically fuel Fenton reactions, propagating lipid peroxidation and ferroptosis^[26,33]^. Our data reveal that BDE47-induced ferroptosis depends on both excessive ROS and Fe^2+^ overload, as antioxidants (*e.g.*, NAC) and iron chelators (*e.g.*, DFP) rescued cell death. Even in H_2_O_2_-treated cells, we detected ferrous ion accumulation, suggesting that ROS may indirectly influence iron homeostasis through mechanisms warranting further investigation. Excessive ROS generated by CYP3A metabolism can modify key signal transduction proteins^[34]^. Therefore, ROS produced during CYP3A-mediated metabolism are essential for the full execution of ferroptosis.

Ferritinophagy is a focal point in recent research on the toxic effects of exogenous chemicals. Thus, we further elucidated how CYP3A-generated ROS triggers ferritinophagy. CYP3A substrates promoted autophagosome formation while suppressing ferritin expression. This process critically depends on autophagy-related (ATG) proteins^[35]^. Our data revealed significant upregulation of ATG12, a ubiquitin-like protein that forms a critical conjugate with ATG5 through sequential activation by ATG7 (E1) and ATG10 (E2) enzymes^[36]^. Although ATG5/ATG7 have been implicated in ferroptosis^[20]^, our study provides the first evidence for the involvement of ATG12 in ferritinophagy using an *Atg12*-haploinsufficiency mouse model. However, knockout of *Atg12* is embryonic lethal in mice, which necessitated the use of *Atg12*^+/^^−^ heterozygous mice in this study. A limitation of this approach is that heterozygous mice exhibit only an approximately 50% reduction in ATG12 expression, which may not fully recapitulate the phenotype that would be observed with complete loss of ATG12 function. It is possible that a more severe or distinct phenotype might occur under conditions of complete ATG12 deficiency, but this cannot be evaluated due to embryonic lethality. Future studies using conditional or tissue-specific *Atg12* knockout models would help to further elucidate the full role of ATG12 in BDE47-induced autophagy and germ cell injury.

Given that the antioxidant effectively attenuated BDE47-induced ATG12 upregulation, we uncovered a new mechanistic link between ROS and ATG12 induction. Our data revealed that ROS promotes the overexpression of HIF-1α, a transcription factor intricately linked to mitochondrial metabolism and ROS generation^[37]^. Previous studies have reported that mitochondrial ROS enhances HIF-1α stability^[38]^, and HIF-1α has been identified as a transcriptional regulator of the m^6^A demethylase ALKBH5, a key m^6^A eraser^[25]^. As a major form of RNA modification, m^6^A is tightly regulated by methyltransferases and demethylases and profoundly influences RNA fate by altering secondary structure, stability, and protein binding^[39]^. m^6^A modification plays important roles in regulating mRNA stability and protein translation, which are involved in various human diseases^[40]^. To assess whether ALKBH5 induction impacts m^6^A dynamics, we demonstrated significant reductions in total m^6^A modification and reduced m^6^A modification at specific sites on *Atg12* mRNA, correlating with its increased mRNA expression, concomitant with elevated ALKBH5 expression. It has been reported that the demethylase FTO promotes the expression of autophagic proteins ATG5 and ATG7 in an m^6^A-dependent manner, thereby affecting adipogenesis, and that there is crosstalk between m^6^A modification and autophagy^[41]^. Our findings suggest that reduced m^6^A modification is associated with increased ATG12 expression; however, whether this effect is mediated by altered *Atg12* mRNA stability requires further investigation. Future studies using m^6^A site-directed mutagenesis or transcriptome-wide m^6^A mapping are required to validate whether *Atg12* is a direct target of ALKBH5-mediated m^6^A demethylation and to dissect the precise molecular mechanism.

**Figure 10:**
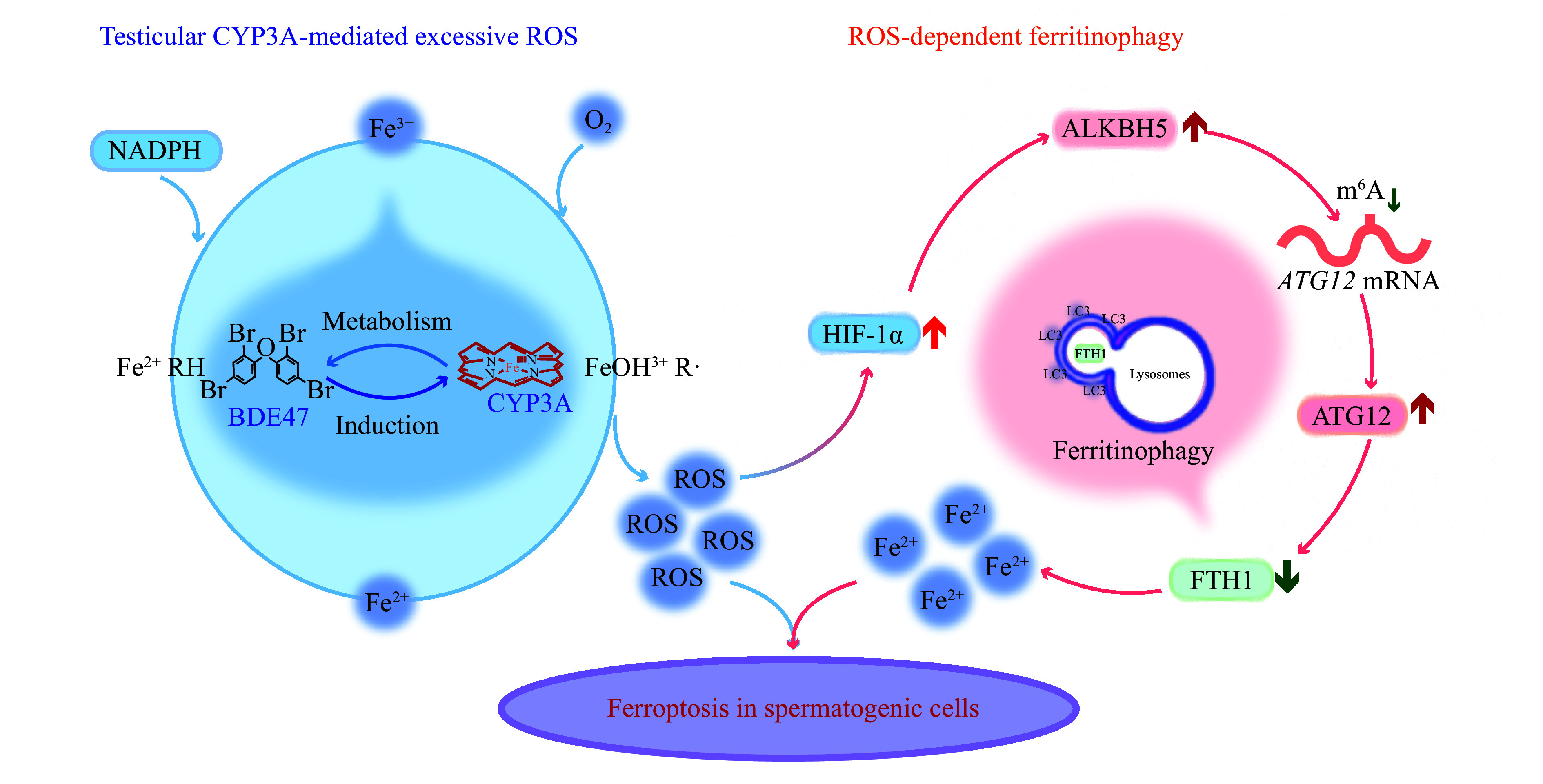
Mechanism diagram of BDE47-induced testicular spermatogenic injury. BDE47 triggers CYP3A expression, causing excessive ROS production. ROS reduces m^6^A methylation of *ATG12* mRNA and increases ATG12 expression, which is associated with increased expression of HIF-1α HIF-1a and ALKBH5. Elevated ATG12 drives autophagic degradation of FTH1, further exacerbating Fe^2+^ overload, while the synergistic interplay between Fe^2+^ and ROS amplifies lipid peroxidation. Abbreviations: ALKBH5, AlkB homolog 5; ATG12, autophagy-related 12; BDE47, 2,2',4,4'-tetrabromodiphenyl ether; CYP3A, cytochrome P450 3A; FTH1, ferritin heavy chain 1; HIF-1α, hypoxia-inducible factor 1α; m^6^A, N6-methyladenosine; ROS, reactive oxygen species.

Our work highlights the role of ROS generated during CYP3A metabolism in BDE47-induced toxicity and clarifies that the metabolic process may be an important etiological factor in reproductive damage caused by xenobiotics, providing a new perspective for explaining the reproductive toxicity of exogenous chemicals (***Fig. 10***). Moreover, mouse *Cyp3a11* was used *in vivo* and human *CYP3A4* *in vitro*, with 72.6% amino acid homology between the two enzymes. The sequence homology and functional effects observed with human *CYP3A4* overexpression suggest that CYP3A-mediated mechanisms may also be relevant to humans. However, species-specific differences in CYP3A expression, substrate metabolism, and testicular physiology preclude direct extrapolation of these findings to human reproductive toxicity. Furthermore, given that *Gpx4* and *Cyp3a11* are expressed in multiple testicular cell types, the precise cell-type specificity of BDE47-induced damage remains to be established. Therefore, our conclusions are limited to indicating that BDE47 causes damage to germ cells, with a predominant but not exclusive effect on spermatocytes based on histopathological observations and previous literature. In addition, mechanistic intervention experiments were largely limited to GC-2 cells, and direct *in vivo* intervention targeting CYP3A in the testes was not performed. Further studies are therefore needed to validate the CYP3A-mediated ferritinophagy pathway *in vivo*. Future research utilizing cell-type-specific knockout or lineage tracing approaches will help to strengthen the conclusions of this study.

## Additional information

The online version contains supplementary materials available at http://www.jbr-pub.org.cn/article/doi/10.7555/JBR.40.20260159.
