# Supplementary material for: CYP3A triggers BDE47-induced ferritinophagy and ferroptosis in spermatogenic cells through ROS-mediated m6A regulation of ATG12
Source: J Biomed Res. 2026 Jul 28;40(4):364–82. doi: 10.7555/JBR.40.20260159 (PMC13438320; doi:10.7555/JBR.40.20260159)
Supplement: Supplementary file 1 [file jbr-40-4-364-S1.pdf]

Available online at www.jbr-pub.org.cn

Open Access at PubMed Central

Journal of Biomedical Research, 2026 40(4): 1–3

Supplementary Data

JBR

CYP3A triggers BDE47-induced ferritinophagy and ferroptosis in spermatogenic cells through ROS-mediated m⁶A regulation of ATG12

Shuyu Xu[1,2,△], Wenxia Fan[2,△], Jiangxue Qian[2], Yuewen He[1,2], Chunjin Li[1,2], Chao Chen[2], Li Wang[2], Yongquan Yu[2], Chao Wang[2,✉], Shoulin Wang[1,2,✉]

[1]*State Key Lab of Reproductive Medicine and Offspring Health, Institute of Toxicology, Nanjing Medical University, Nanjing, Jiangsu 211116, China;*

[2]*Key Lab of Modern Toxicology of Ministry of Education, Center for Global Health, School of Public Health, Nanjing Medical University, Nanjing, Jiangsu 211116, China.*

Supplementary Table 1 **List of antibodies used in this study**

Antibodies	Brand	Cat. No.	Applications
ALKBH5	Proteintech	16837-1-AP	WB (1 : 1 000)
ATG12	Cell signal	4180P	WB (1 : 1 000)
ATG5	Cell signal	12994P	WB (1 : 1 000)
ATG7	Cell signal	8558P	WB (1 : 1 000)
CYP3A4	Proteintech	18227-1-AP	WB (1 : 1 000); IF (1 : 100)
FTH1	Afantibody	AF300646	WB (1 : 1 000); IF (1 : 100)
FTO	Proteintech	27226-1-AP	WB (1 : 1 000)
GAPDH	Proteintech	10494-1-AP	WB (1 : 1 000)
Goat Anti-Mouse IgG (H + L) HRP Secondary Antibody	OriGene	TA373082	WB (1 : 5 000)
Goat Anti-Rabbit IgG (H + L) Alexa Fluor 594	Abways	AB0151	IF (1 : 200)
Goat Anti-Rabbit IgG (H + L) HRP Secondary Antibody	OriGene	TA373083	WB (1 : 2 000)
GPX4	Beyotime	AF7020	WB (1 : 1 000)
HIF-1α	Abcam	ab179483	WB (1 : 1 000); ChIP-qPCR
LC3B-β	Cell signal	12741T	WB (1 : 1 000); IF (1 : 100)
m⁶A	Invitrogen	MA5-33030	Dot blotting; MeRIP-qPCR
METTL14	Proteintech	26158-1-AP	WB (1 : 1 000)
METTL3	Afantibody	AF302106	WB (1 : 1 000)
NCOA4	Affinity	DF4255	WB (1 : 1 000); IF (1 : 100)
WTAP	Cell signal	56501T	WB (1 : 1 000)

△These authors contributed equally to this work.

✉Corresponding authors: Shoulin Wang, E-mail: wangshl@njmu.edu.cn. ORCID: 0000-0003-3070-7560; Chao Wang, E-mail: wangchao@njmu.edu.cn, ORCID: 0000-0001-8452-1214.

Received: 18 March 2026; Revised: 12 May 2026; Accepted: 14 May 2026; Available online: 21 May 2026; Published date: 28 July 2026

CLC number: R114; R698.2, Document code: A

The authors reported no conflicts of interest.

This is an open access article under the Creative Commons Attribution (CC BY 4.0) license, which permits others to distribute, remix, adapt and build upon this work, for commercial use, provided the original work is properly cited.

© 2026 by Journal of Biomedical Research.

https://doi.org/10.7555/JBR.40.20260159

Supplementary Table 2 **Protein sequences for human *CYP3A4* and mouse *Cyp3a11***

Protein names	Amino acid sequence
Human *CYP3A4*	MALIPDLAMETWLLLAVSLVLLYLYGTHSHGLFKKLGIPGPTPLPF<br>LGNILSYHKGFCMFDMECHKKYGKVWGFYDGQQPVLAITDPDMI<br>KTVLVKECYSVFTNRRPFGPVGFMKSAISIAEDEEWKRLRSLLSPTF<br>TSGKLKEMVPIIAQYGDVLVRNLRREAETGKPVTLKDVFGAYSMD<br>VITSTSFGVNIDSLNNPQDPFVENTKKLLRFDFLDPFFLSITVFPFLIPI<br>LEVLNICVFPREVTNFLRKSVKRMKESRLEDTQKHRVDFLQLMIDS<br>QNSKETESHKALSDLELVAQSIIFIFAGYETTSSVLSFIMYELATHPD<br>VQQKLQEEIDAVLPNKAPPTYDTVLQMEYLDMVVNETLRLFPIAM<br>RLERVCKKDVEINGMFIPKGVVVMIPSYALHRDPKYWTEPEKFLPE<br>RFSKKNKDNIDPYIYTPFGSGPRNCIGMRFALMNMKLALIRVLQNF<br>SFKPCKETQIPLKLSLGGLLQPEKPVVLKVESRDGTVSGA
Mouse *Cyp3a11*	MDLVSALSLETWVLLAISLVLLYRYGTRKHELFKKQGIPGPKPLPF<br>LGTVLNYYKGLWKFDMECYKKYGKTWGLFDGQTPLLAVTDPETI<br>KNVLVKECFSVFTNRRDFGPVGIMSKAISISKDDEWKRYRALLSPT<br>FTSGKLKEMFPVIEQYGDILVKYLRQKAKKGKPVTMKDVLGAYS<br>MDVITSTSFGVNVDSLNNPEDPFVEKAKKLLRFDFFDPLLFSVVLFP<br>FLTPVYEMLNICMFPKDSIEFFKKFVDRMKESRLDSKQKHRVDFLQ<br>LMMNSHNNSKDKVSHKALSDMEITAQSIIFIFAGYETTSSTLSFTLH<br>SLATHPDIQKKLQDEIDEALPNKAPPTYDTVMEMEYLDMVLNETL<br>RLYPIANRLERVCKKDVELNGVYIPKGSTVMIPSYALHHDPQHWS<br>EPEEFQPERFSKENKGSIDPYVYLPFGNGPRNCLGMRFALMNMKL<br>ALTKIMQNFSFQPCKETQIPLKLSRQGLLQPEKPIVLKVVPRDAVIT GA

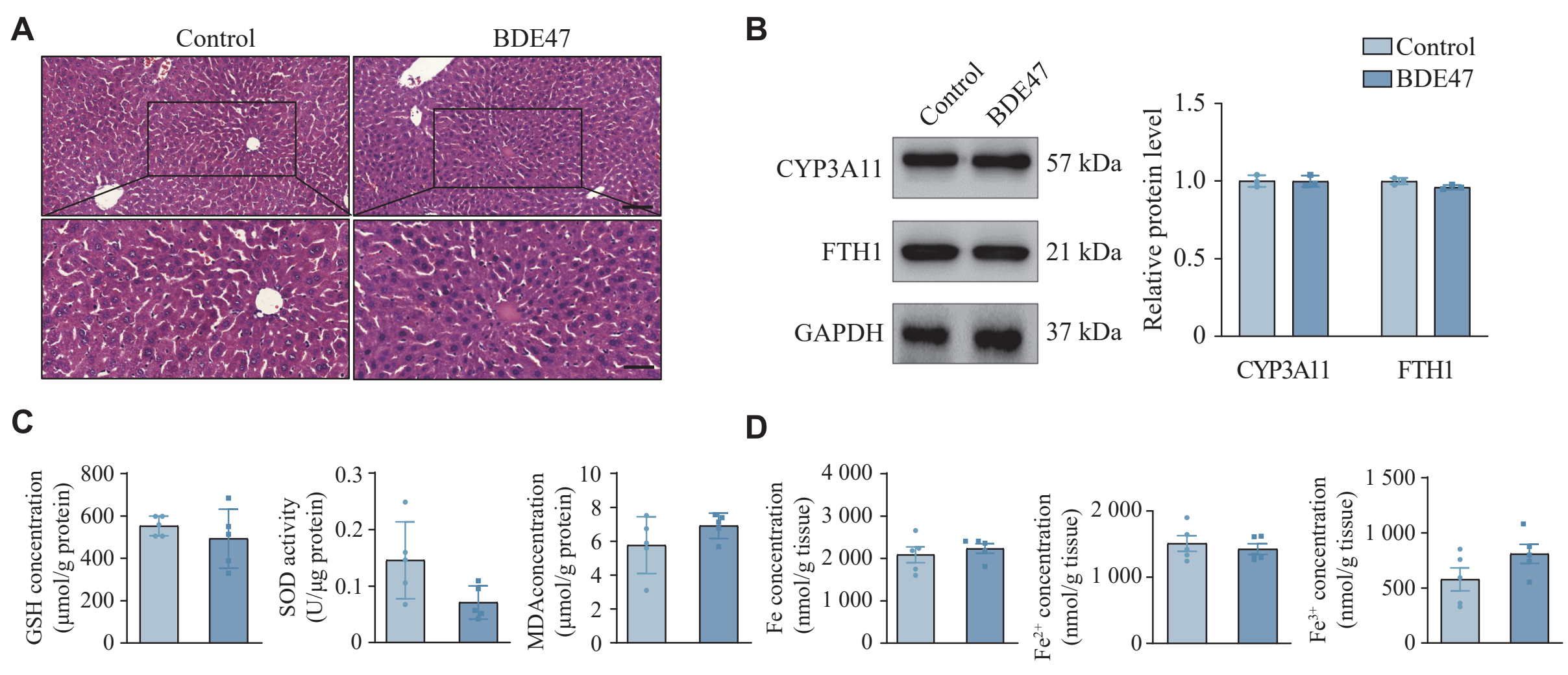

Supplementary Fig. 1 **Effects of BDE47 on oxidative stress and ferroptosis in mouse liver.** Male C57BL/6 mice (n = 5 per group) were treated with BDE47 (1.5 mg/kg) by oral gavage for 8 weeks; the control group received saline with an equal amount of DMSO. A: Histopathological observation of mouse liver tissues with H&E staining; magnified views are shown below. Scale bars: 50 μm and 100 μm, respectively. B: Expression of CYP3A11 and FTH1 in mouse liver tissue was detected using Western blotting. GAPDH was used as a loading control. C: Determination of GSH, SOD, and MDA levels in liver tissue using colorimetric assay kits. D: Iron content (total Fe, Fe^{2+}, and Fe^{3+}) in mouse liver tissue measured using a colorimetric assay kit. All data are presented as mean ± standard deviation. Statistical analysis was performed using Student's t-test. $^{*}P < 0.05$. Abbreviations: BDE47, 2,2',4,4'-tetrabromodiphenyl ether; CYP3A11, cytochrome P450 family 3 subfamily A member 11; FTH1, ferritin heavy chain 1; GSH, glutathione; MDA, malondialdehyde; SOD, superoxide dismutase.

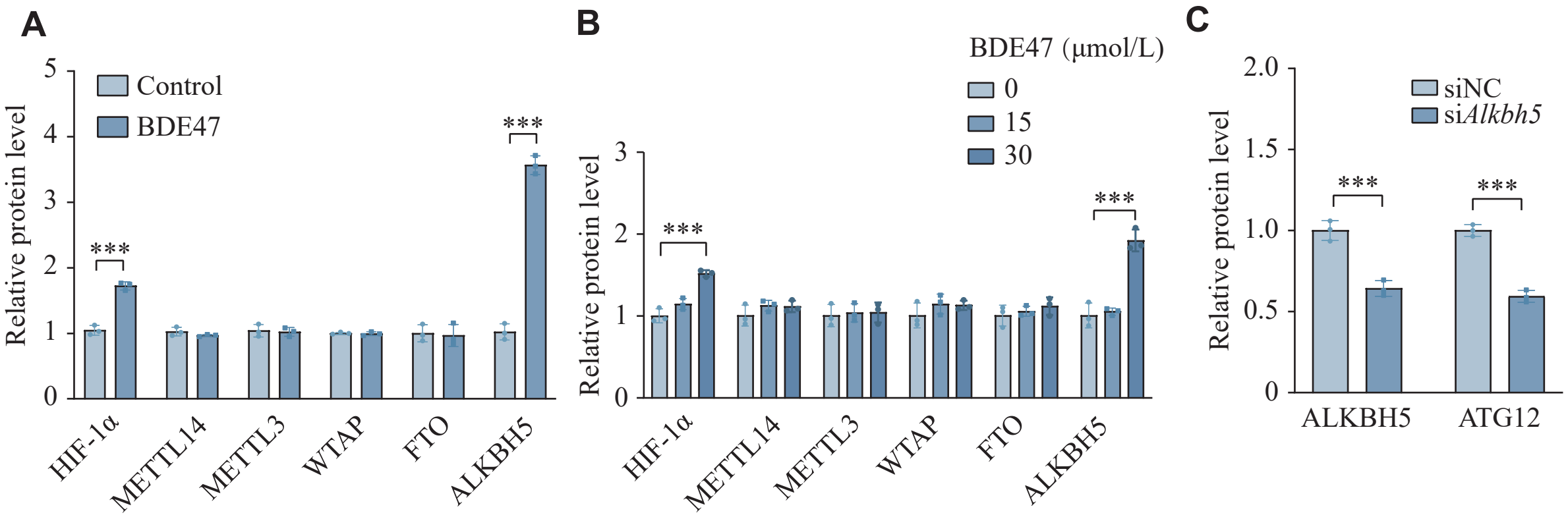

Supplementary Fig. 2 **Quantification of related protein intensity in *Fig. 5*.** Quantitative analyses of Western blotting results (***Fig 5A***, ***5B***, and ***5J***) in testicular tissues of mice (A), GC-2 cells (B), and the si*Alkbh5* interference experiment (C). Statistical analysis was performed using Student's *t*-test (A and C) or one-way ANOVA with Tukey's post hoc test (B). $^{***}P < 0.001$.
